# Regulatory subunit NEMO promotes polyubiquitin-dependent induction of NF-κB through a targetable second interaction with upstream activator IKK2

**DOI:** 10.1016/j.jbc.2022.101864

**Published:** 2022-03-24

**Authors:** Myung Soo Ko, Samantha N. Cohen, Smarajit Polley, Sushil K. Mahata, Tapan Biswas, Tom Huxford, Gourisankar Ghosh

**Affiliations:** 1 Department of Chemistry & Biochemistry, University of California, San Diego, La Jolla, California, USA; 2 Structural Biochemistry Laboratory, Department of Chemistry & Biochemistry, San Diego State University, San Diego, California, USA; 3 Department of Medicine, University of California, San Diego, La Jolla, California, USA; 4 Medicine, VA San Diego Health Care System, San Diego, California, USA

**Keywords:** enzyme inactivation, NF-κB, peptide interaction, polyubiquitin chain, protein kinase, BMDM, bone marrow–derived macrophage, CC2, coiled-coil 2, cDNA, complementary DNA, DMEM, Dulbecco's modified Eagle's medium, Erk2, extracellular signal–regulated kinase 2, GST, glutathione-*S*-transferase, HA, hemagglutinin, HEK293T, human embryonic kidney 293T cell line, IKK, inhibitor of κB kinase, IL, interleukin, JNK, c-Jun N-terminal kinase, KBD, kinase-binding domain, KD, kinase domain, LPS, lipopolysaccharide, LTβR, lymphotoxin-β receptor, MAPK, mitogen-activated protein kinase, MEF, mouse embryonic fibroblast, MIP, macrophage inflammatory protein, NBD, NEMO-binding domain, NEMO, NF-κB essential modulator, NEMO^ActPep^, NEMO activation peptide, NTA, nitrilotriacetic acid, RIPA, radioimmunoprecipitation assay, SDD, scaffold-dimerization domain, SEC, size-exclusion chromatography, TEV, tobacco etch virus, TNF-α, tumor necrosis factor alpha, Ub, ubiquitin, ULD, ubiquitin-like domain

## Abstract

Canonical NF-κB signaling through the inhibitor of κB kinase (IKK) complex requires induction of IKK2/IKKβ subunit catalytic activity *via* specific phosphorylation within its activation loop. This process is known to be dependent upon the accessory ubiquitin (Ub)-binding subunit NF-κB essential modulator (NEMO)/IKKγ as well as poly-Ub chains. However, the mechanism through which poly-Ub binding serves to promote IKK catalytic activity is unclear. Here, we show that binding of NEMO/IKKγ to linear poly-Ub promotes a second interaction between NEMO/IKKγ and IKK2/IKKβ, distinct from the well-characterized interaction of the NEMO/IKKγ N terminus to the “NEMO-binding domain” at the C terminus of IKK2/IKKβ. We mapped the location of this second interaction to a stretch of roughly six amino acids immediately N-terminal to the zinc finger domain in human NEMO/IKKγ. We also showed that amino acid residues within this region of NEMO/IKKγ are necessary for binding to IKK2/IKKβ through this secondary interaction *in vitro* and for full activation of IKK2/IKKβ in cultured cells. Furthermore, we identified a docking site for this segment of NEMO/IKKγ on IKK2/IKKβ within its scaffold-dimerization domain proximal to the kinase domain–Ub-like domain. Finally, we showed that a peptide derived from this region of NEMO/IKKγ is capable of interfering specifically with canonical NF-κB signaling in transfected cells. These *in vitro* biochemical and cell culture–based experiments suggest that, as a consequence of its association with linear poly-Ub, NEMO/IKKγ plays a direct role in priming IKK2/IKKβ for phosphorylation and that this process can be inhibited to specifically disrupt canonical NF-κB signaling.

Mobilization of transcription factor NF-κB to the nucleus in response to diverse proinflammatory stimuli requires phosphorylation of activation loop serines 177 and 181 within the inhibitor of κB kinase (IKK)2/IKKβ subunit of the IKK complex (1). The IKK complex also contains catalytic IKK1/IKKα and accessory NF-κB essential modulator (NEMO)/IKKγ subunits (hereafter referred to as IKK2, IKK1, and NEMO) (2, 3, 4). Upon activation, the catalytic IKK2 subunit directs site-specific phosphorylation of the IκBα inhibitor protein, leading to its ubiquitin (Ub)-dependent degradation *via* the 26S proteasome and release of the classical NF-κB p50–RelA heterodimer, which migrates into the nucleus to direct response gene expression (5, 6). As illustrated by gene knockout studies, the NEMO subunit of the IKK complex is required for induction of NF-κB (7, 8). Moreover, before the IKK complex had even been identified unambiguously, it was shown that stimulation of IKK catalytic activity from partially purified cell lysates requires both Ub and ATP (9). Ub assembles into K63-linked and M1-linked linear poly-Ub chains in response to early NF-κB signaling events (10, 11). Linear poly-Ub chains associate both covalently and noncovalently with NEMO; however, the noncovalent interaction has been proven to be sufficient for induction of NF-κB transcriptional activity through the canonical signaling pathway (12, 13).

Three-dimensional structures of free IKK2 and IKK1 have revealed that they adopt similar structural folds (14, 15, 16, 17). Both catalytic domain–containing IKK subunits assemble in solution as homodimers. Interestingly, both IKK2 and IKK1 exhibit a strong propensity for higher degree oligomerization through ordered self-association, although the precise nature of the oligomerization differs significantly between the two proteins (15, 17). Despite their 50% amino acid sequence identity and 80% sequence homology, IKK2 and IKK1 rely upon unique surface-exposed regions to mediate different higher order assemblies in order to render their activation loops accessible for transphosphorylation. In light of these observations, we previously proposed a model for induction of IKK catalytic potential *via* activation loop phosphorylation as a consequence of stabilizing catalytic IKK subunit dimers in their “open” conformation (4, 15). Under such a mechanism, it is unclear whether the necessary role of NEMO is that of an adaptor that simply colocalizes catalytic subunits through poly-Ub chains to sites where homo-oligomerization can promote activation loop phosphorylation or if NEMO–poly-Ub complexes play a more direct role in facilitating IKK2 subunit phosphorylation and consequent catalytic activity.

In this study, we provide evidence that, upon noncovalent binding to linear poly-Ub, NEMO directly promotes activation loop phosphorylation of the catalytic IKK2 subunit. We identify a second interaction between NEMO and IKK2 that is dependent upon NEMO binding to linear poly-Ub. We map this newly identified NEMO–IKK2 interaction interface to a stretch of six conserved amino acids immediately N-terminal to the Zn-finger domain at the C terminus of human NEMO and an exposed region of the IKK2 scaffold-dimerization domain (SDD) proximal to its kinase domain (KD) and ubiquitin-like domain (ULD). A peptide derived from the second interaction interface of NEMO serves to inhibit transphosphorylation of the IKK2 subunit *in vitro* and blocks canonical NF-κB signaling in cell culture.

## Results

### Linear poly-Ub and NEMO prime IKK2 for transphosphorylation *in vitro*

**Figure 1:**
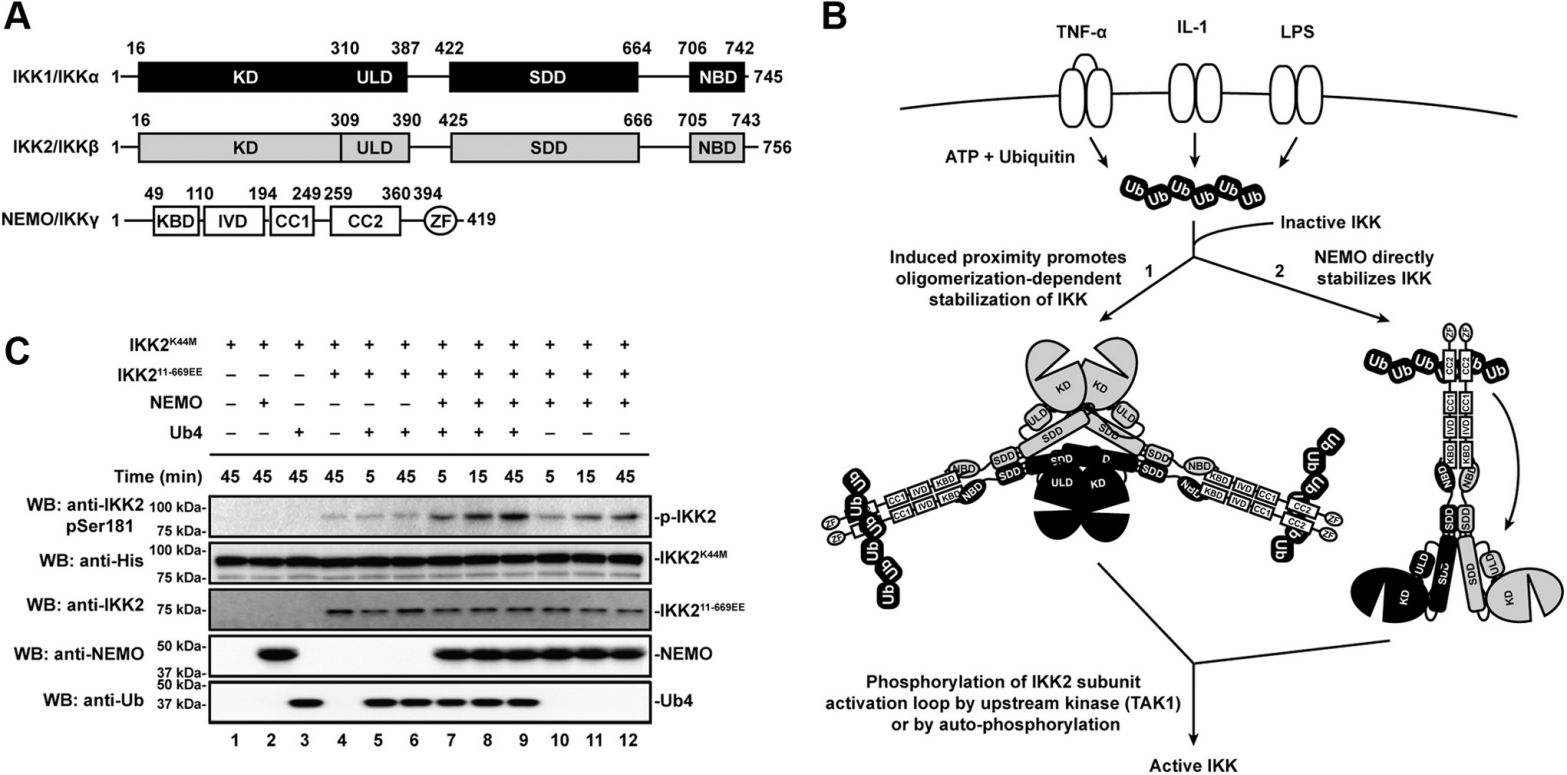
**NEMO primes IKK2 for transphosphorylation on its activation loop in the presence of linear polyubiquitin.***A*, schematics of domains for human IKK1/IKKα, IKK2/IKKβ, and NEMO/IKKγ protein subunits. Both IKK1 and IKK2 contain a well-defined kinase domain (KD) as well as an ubiquitin-like domain (ULD), a scaffold dimerization domain (SDD), and a NEMO-binding domain (NBD). Structurally and/or functionally defined domains of NEMO include a kinase-binding domain (KBD), intervening domain (IVD), coiled-coil domains 1 and 2 (CC1 and CC2), and a zinc finger (ZF) domain. *B*, alternative possible modes of NEMO and polyubiquitin control over IKK2 subunit phosphorylation within the canonical NF-κB signaling pathway. In the first (depicted symbolically on the left fork labeled “1”), polyubiquitin chains generated as a result of proinflammatory cytokine engagement with receptors serve to localize IKK subunits on account of their affinity for NEMO adaptor proteins. A second possibility (right fork labeled “2”) is that polyubiquitin binding enables NEMO to directly influence the structural conformation of IKK2. In either case, the result is stabilization of the IKK complex in a conformation that permits transphosphorylation of the IKK2 subunit activation loop and IKK activity. *C*, Western blot analysis of *in vitro* transphosphorylation of a catalytically inactive form of IKK2 (K44M) by a constitutively active IKK2 (11–669EE). Inclusion of NEMO and linear tetraubiquitin (Ub4) improves efficiency of phosphorylation (lanes 7–9) relative to either Ub4 (lanes 5 and 6) or NEMO (lanes 10–12) alone. IKK, inhibitor of κB kinase; NEMO, NF-κB essential modulator.

NEMO, IKK1, and IKK2 associate noncovalently within the IKK complex under physiological conditions, and both NEMO and IKK2 are required for stimulus-dependent response gene expression through the canonical NF-κB signaling pathway (Fig. 1, *A* and *B*) (18). The mechanism underlying the obligatory role of NEMO in promoting IKK2 catalytic activity is unclear. In light of previous observations that catalytic IKK subunits are prone to spontaneous ordered oligomerization and that, as a consequence of these stabilizing interactions, IKK can undergo phosphorylation-dependent activation *in vitro* at even moderately higher than cellular concentrations independent of NEMO, we imagined that NEMO might play a passive adaptor role. Under this mechanism for oligomerization-dependent transphosphorylation, which was first proposed by Häcker and Karin in 2006 (19), linear poly-Ub could serve as an anchoring scaffold to recruit and localize multisubunit IKK complexes to intracellular signaling assemblies. Alternatively, it seems plausible that NEMO might participate directly in priming IKK2 for activation loop phosphorylation and catalytic activity in response to binding linear poly-Ub (Fig. 1*B*). Such a mechanism has been suggested by Rahighi *et al.* (20).

To directly test this second hypothesis, we examined *in vitro* with purified recombinant proteins if accessibility of the IKK2 activation loop to protein kinase transphosphorylation relies upon the binding of linear poly-Ub and NEMO. To this end, we employed a catalytically inactive version of human IKK2 (IKK2^K44M^) that contains an otherwise native sequence, unphosphorylated activation loop, and, therefore, serves exclusively as a substrate for activation loop transphosphorylation (Fig. S1*A*) (15). To this substrate was added a constitutively active version of human IKK2 with both its key activation loop serines (Ser177 and Ser181) mutated to phosphomimetic glutamic acids and its C-terminal NEMO-binding domain (NBD) deleted (IKK2^11–669EE^) at a substrate:enzyme ratio of 2 μg:30 ng. Activation loop phosphorylation was monitored *via* anti-pSer181 Western blot for the substrate IKK2^K44M^. Linear poly-Ub chains bind to the coiled-coil 2 (CC2) domain, also known as the “CoZi,” of NEMO (Fig. 1*A*) (20, 21). Therefore, to assess the effect of linear poly-Ub binding to NEMO on IKK2 activation loop phosphorylation, we next added recombinant human NEMO alone or in combination with linear tetra-Ub (Ub4) chains generated as four N-terminal to C-terminal covalently linked Ub moieties (M1 linked). Recombinant Ub4 associates with NEMO *in vitro* as evidenced by size-exclusion chromatography (SEC) (Fig. S1*B*). Furthermore, Ub4 was deemed as likely sufficient to actuate an effect on IKK2 activation loop phosphorylation since M1-linked di-Ub is known to bind NEMO *in vitro* and has been demonstrated to trigger efficient NF-κB activation in cells (22).

We observe by Western blot that the IKK2 activation loop remains largely unphosphorylated in the presence of constitutively active kinase (Fig. 1*C*). Activation loop phosphorylation increased significantly after reactions were supplemented with NEMO and was enhanced to an even greater extent upon inclusion of both NEMO and Ub4. In the absence of NEMO, Ub4 had no impact on phosphorylation of the IKK2 activation loop. These results suggest that linear poly-Ub chain binding to NEMO serves to prime the activation loop of IKK2 such that it is amenable to phosphorylation in *trans* at Ser177 and Ser181.

### Linear poly-Ub induces a novel interaction of NEMO and IKK2 through a “second” site

In light of our observation that NEMO efficiently primes the IKK2 activation loop for transphosphorylation in the presence of linear poly-Ub, we hypothesized that, in addition to the well-characterized high-affinity interaction between the N-terminal segment of NEMO known as the kinase-binding domain (KBD) and the C-terminal NBD of IKK2, poly-Ub binding might promote additional intermolecular interactions involving other regions of NEMO and IKK2 (23, 24, 25). Such poly-Ub–dependent additional interactions could stabilize IKK2 in its open conformation, which is known to support activation loop phosphorylation in *trans* either *via* autophosphorylation by IKK2 itself or by other upstream kinases such as transforming growth factor-β–activated kinase 1 (26).

**Figure 2:**
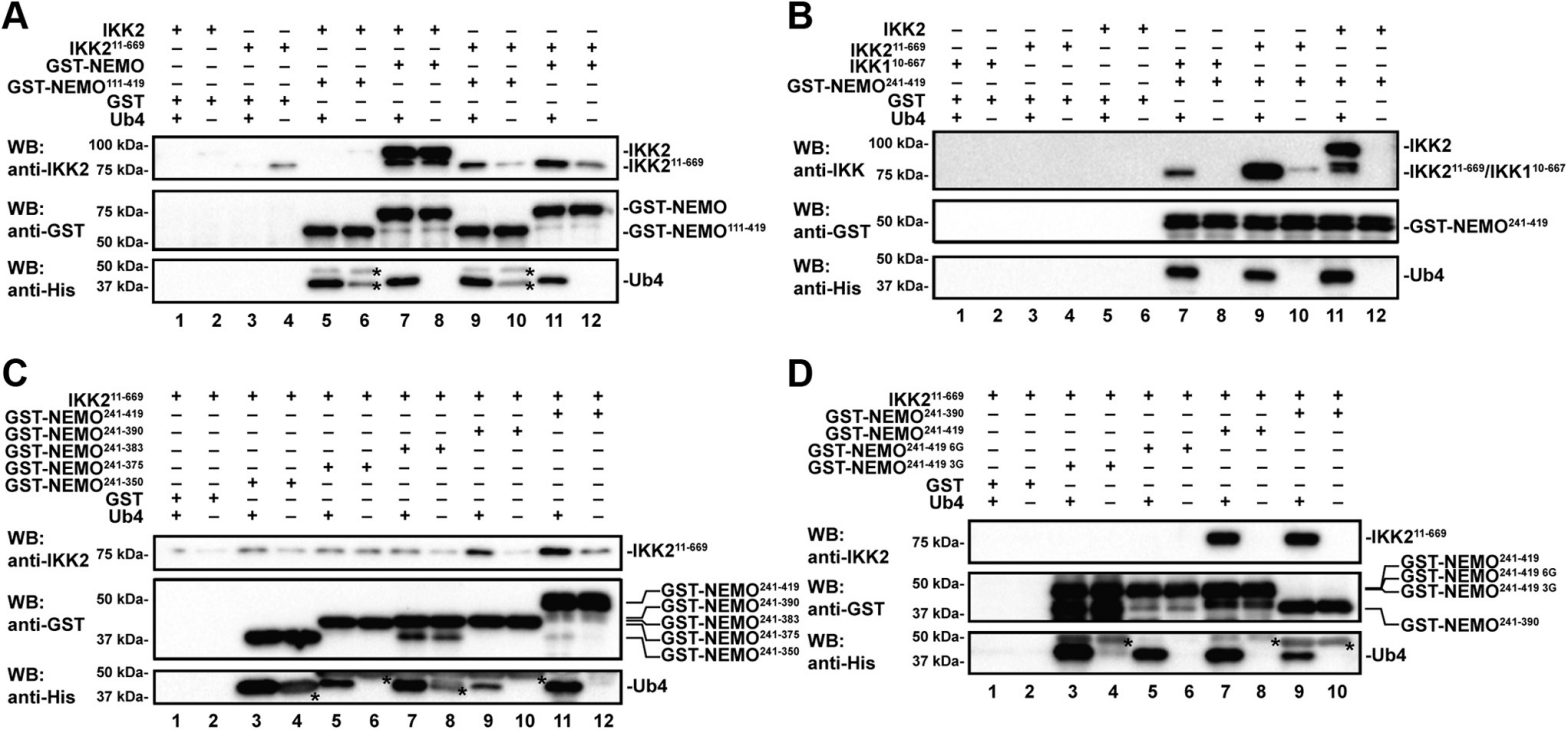
**NEMO mediates a secondary interaction with IKK2 upon interaction with linear polyubiquitin.***A*, Western analysis of *in vitro* GST pull down with purified recombinant IKK2 and NEMO. Primary antibodies are listed on the *left* of each panel, and the presence of each protein is indicated *above* each lane. Full-length NEMO binds full-length IKK2 (lanes 7 and 8), whereas removal of the NBD results in NEMO–IKK2 binding that is dependent upon the presence of linear polyubiquitin (Ub4) (lanes 9–12). *B*, the C-terminal CC2-ZF (NEMO^241–419^) is sufficient to bind IKK2 in the presence of Ub4 (lanes 9–12) but interacts only very weakly with IKK1 (lanes 7 and 8). *C*, removal of the C-terminal ZF (NEMO^241–390^) does not affect Ub4-dependent binding to IKK2 (lanes 9 and 10) while further deletion of NEMO residues 384 to 390 (NEMO^241–383^) disrupts the interaction (lanes 3–8). *D*, mutation of NEMO residues 384 to 389 (lanes 5 and 6) or 384 to 386 (lanes 3 and 4) is sufficient to disrupt the Ub4-dependent secondary binding of NEMO to IKK2. *Asterisks* in the *bottom panels* of *A*, *C*, and *D* indicate proteins from the GST-NEMO preparations that, on account of the relatively high amount of GST-NEMO required to pull-down IKK2 *via* secondary interaction, react nonspecifically with the anti-His primary antibody. CC2, coiled-coil 2; GST, glutathione-*S*-transferase; IKK, inhibitor of κB kinase; NBD, NEMO-binding domain; NEMO, NF-κB essential modulator; ZF, zinc finger.

We produced multiple variants of human NEMO lacking its N-terminal KBD (amino acids 1–110) and assessed the abilities of these NEMO variants to interact with human IKK2 variants lacking their C-terminal NBD (amino acids 670–756) using *in vitro* affinity pull-down experiments both in the presence and absence of a linear poly-Ub (Fig. 2). Full-length and truncated NEMO were expressed in *Escherichia coli* and purified to homogeneity as glutathione-*S*-transferase (GST)-fusion proteins (Fig. S2*A*). Both full-length and truncated (amino acids 111–419) NEMO proteins exhibited association with Ub4, as expected because of their intact CC2 domain (Fig. 2*A*, lanes 5 and 7; Fig. S3). Full-length NEMO and full-length IKK2 displayed a strong interaction, as expected on account of the well-characterized interaction of the IKK2 NBD and NEMO KBD, and the presence of Ub4 did not result in any discernible change to this highly stable complex. Interestingly, the experiment revealed an interaction between IKK2^11–669^ (lacking the NBD) and either full-length NEMO or NEMO^111–419^ (lacking the KBD) that was enhanced in the presence of Ub4 (Fig. 2*A*; lanes 9–12). This observation is consistent with an additional NEMO–IKK2 interaction that is dependent upon the poly-Ub–binding status of NEMO. Surprisingly, we do not observe pull down of full-length IKK2 by GST-NEMO^111–419^ in the absence or the presence of Ub4. This suggests that the native C-terminal NBD of IKK2 interferes with the ability of NEMO to mediate a second interaction in the absence of its binding with the NEMO N-terminal KDB.

To define this second interaction more precisely, we investigated the region of NEMO that mediates Ub4 binding (20, 21). We first generated GST-NEMO^241–419^, lacking its N-terminal KBD, intervening domain, and CC1 regions, and validated its ability to bind Ub4 both by SEC and GST pull-down assays (Figs. 1*A* and S2*B*). Pull-down assays with NEMO^241–419^ and both full-length and truncated IKK2 suggest interactions of near equal efficiency as observed with NEMO^111–419^ in the presence of Ub4 but not in its absence (Fig. 2*B*). The possibility of a similar interaction of NEMO with IKK1 was also investigated. Although an interaction of NEMO with IKK^110–667^ in the presence of Ub4 was detected by GST pull down, it appeared significantly less robust in comparison to that of NEMO and IKK2 (Fig. 2*B*).

To further pinpoint the region(s) of NEMO involved in the observed second interaction, we generated NEMO^241–390^, NEMO^241–383^, NEMO^241–375^, and NEMO^241–350^ as GST–fusion proteins and, consistent with each of the NEMO fragments containing their linear poly-Ub–binding CC2 region, confirmed their ability to bind Ub4 by SEC (Figs. 1*A* and S2*C*). Pull-down experiments in the presence of Ub4 indicated that both GST-NEMO^241–390^ and GST-NEMO^241–419^ are capable of interacting with IKK2^11–669^ (Fig. 2*C*; lanes 9–12). This suggests that the C-terminal Zn-finger domain (amino acids 391–419) of NEMO is not required for the second interaction. However, the observed amount of bound IKK2^11–669^ diminished drastically when GST-NEMO^241–383^, GST-NEMO^241–375^, or GST-NEMO^241–350^ were employed in the pull down (Fig. 2*C*, lanes 3–8). Therefore, a small segment encompassing human NEMO residues 383 to 390 appears to be critical for Ub4-dependent binding of IKK2 through a novel second interaction site.

We analyzed the amino acid sequences of NEMO from different mammalian species within this region and observed a strong conservation of a short segment of polypeptide sequence QRRSPP spanning residues 384 to 389 (Fig. S2*D*). To confirm involvement of this small segment in second-site binding, we generated two variants of GST-NEMO^241–419^ in which either all six of these residues or only the first three (residues 384–386) were mutated to glycine (NEMO^241–419 6G^ and NEMO^241–419 3G^, respectively). Affinity pull-down experiments with GST-NEMO^241–419 6G^ or GST-NEMO^241–419 3G^ mutant proteins revealed their almost complete absence of interaction with IKK2 in the presence of Ub4 (Fig. 2*D*). We conclude that NEMO amino acid residues 384 to 389, which reside immediately N-terminal to the Zn-finger domain, are required for the poly-Ub–dependent second interaction between NEMO and IKK2 observed *in vitro*.

### Residues 384 to 389 of human NEMO promote IKK2 activation loop phosphorylation

**Figure 3:**
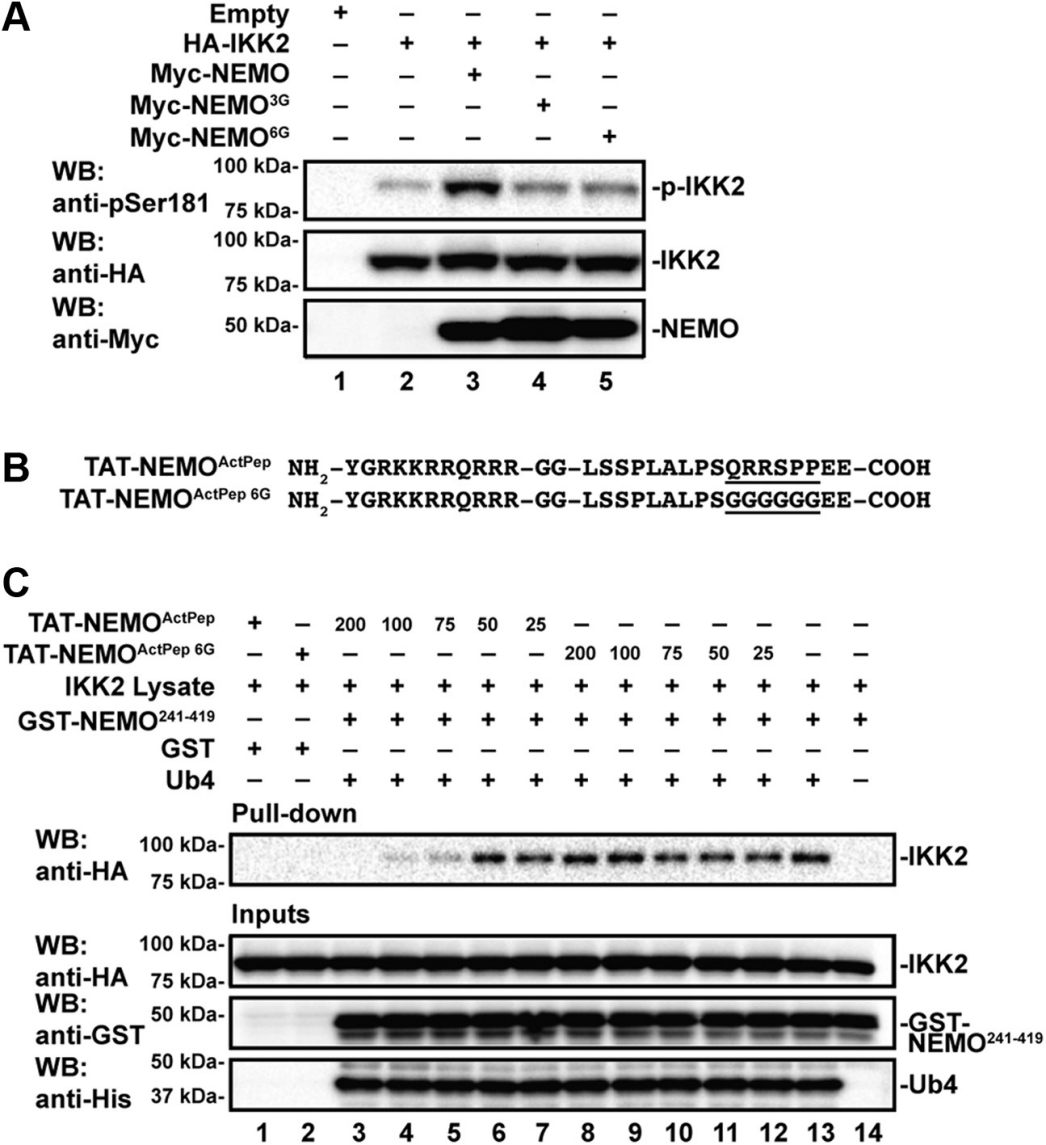
**NEMO residues 384 to 389 are required for full IKK2 activation in cells, and the peptide in isolation competitively inhibits secondary site binding.***A*, Western blot analysis of lysates from HEK293T cells transfected with full-length human HA-IKK2 and full-length human Myc-NEMO of either native sequence or with residues 384 to 386 (3G) or 384 to 389 (6G) mutated to glycines. IKK2 activation loop hyperphosphorylation is observed when both IKK2 and NEMO are overexpressed (*top panel*, lane 3). Mutant NEMO proteins do not support robust IKK2 activation loop hyperphosphorylation (*top panel*, lanes 4 and 5). *B*, amino acid sequences for peptides consisting of HIV-1 TAT fused flexibly to human NEMO amino acids 375 to 391 (TAT-NEMO^ActPep^) as well as a variant with residues 384 to 389 mutated to glycine (TAT-NEMO^ActPep 6G^). *C*, Western blot analysis of GST-pull downs (*top panel*) from HA-IKK2-transfected 293 cell lysates in the presence of linear tetraubiquitin and NEMO^ActPep^ and treated with decreasing (200–25 μM) TAT-NEMO^ActPep^ or TAT-NEMO^ActPep 6G^. The inputs containing lysates augmented with purified proteins and peptides are shown below. GST, glutathione-*S*-transferase; HA, hemagglutinin; HEK293T, human embryonic kidney 293T cell line; IKK, inhibitor of κB kinase; NEMO, NF-κB essential modulator.

Having established that binding to linear poly-Ub induces NEMO to interact with IKK2 through a novel second binding site, we assessed the effect of disrupting this interaction on IKK2 activation in cells. We generated expression plasmids encoding Myc-tagged full-length human NEMO with either residues 384 to 389 (Myc-NEMO^6G^) or 384 to 386 (Myc-NEMO^3G^) mutated to glycines. These plasmids were transfected into cultured human embryonic kidney 293T (HEK293T) cells, and their ability to influence the degree of IKK2 activation was assessed by anti-pSer181 Western blot. Earlier studies have revealed that IKK2, when overexpressed in mammalian cells, becomes partially activated, likely as a consequence of its propensity to oligomerize in a manner that promotes its transphosphorylation (15). The extent of IKK2 activation is further enhanced (hyperactivation) when NEMO is simultaneously overexpressed through cotransfection. We observed typical hyperactivation of IKK2 when it was cotransfected with full-length human Myc-NEMO of native sequence; however, activation was severely diminished with either Myc-NEMO^6G^ or Myc-NEMO^3G^ (Fig. 3*A*). These results strongly suggest a critical role for the NEMO 384 to 389 segment in activation of IKK2 within the cell.

### A peptide derived from NEMO residues 384 to 389 blocks NEMO second-site binding to IKK2

Having observed that the region of NEMO encompassing amino acids 384 to 389 mediates second-site binding to IKK2 and influences IKK2 activation in transfected cells, we explored if an isolated peptide derived from this region could compete with NEMO for its poly-Ub–dependent interaction with IKK2. We designed a heptadecapeptide spanning human NEMO amino acid residues 375 and 391, dubbed “NEMO activation peptide” (NEMO^ActPep^), and prepared a cell permeable version containing an N-terminal HIV-1 TAT sequence (TAT-NEMO^ActPep^). A corresponding control peptide with NEMO residues 384 to 389 altered to glycines (NEMO^ActPep 6G^) was also generated (Fig. 3*B*). We then performed pull-down experiments from extracts of HEK293T cells transfected with plasmids encoding for hemagglutinin (HA)-IKK2 using GST-NEMO^241–419^ alone or in combination with Ub4 and incubated in the absence or the presence of increasing concentrations of TAT-NEMO^ActPep^. As observed with recombinant proteins, GST-NEMO^241–419^ pulled down HA-IKK2 from transfected HEK293T cell lysates only in the presence of Ub4 (Fig. 3*C*, lanes 13 and 14). We observed a loss of this interaction with increasing concentrations of TAT-NEMO^ActPep^ (Fig. 3*C*, lanes 3–7) but not with TAT-NEMO^ActPep 6G^ (Fig. 3*C*, lanes 8–12). Neither peptide appeared to have any effect upon Ub4 binding to NEMO. We conclude that the TAT-NEMO^ActPep^ serves to inhibit the poly-Ub–dependent second interaction between NEMO and IKK2 through competition.

### Identification of a NEMO^ActPep^ docking site on IKK2

Truncated IKK2 proteins encompassing only the KD and ULD are neither regulated properly nor specific toward their substrate IκBα in the absence of the IKK2 SDD (14, 27). The SDD resides adjacent to the KD–ULD and mediates subunit dimerization as well as providing flexibility for movement between the open and closed conformations of the IKK2 and IKK1 homodimers (28). Thus, we tested the possibility that the SDD might participate in the observed linear poly-Ub–dependent second interaction with NEMO through a structure-guided mutagenesis study targeting multiple potential binding residues primarily located within the IKK2 SDD abutting the KD–ULD that has been referred to previously as the “proximal SDD.”

**Figure 4:**
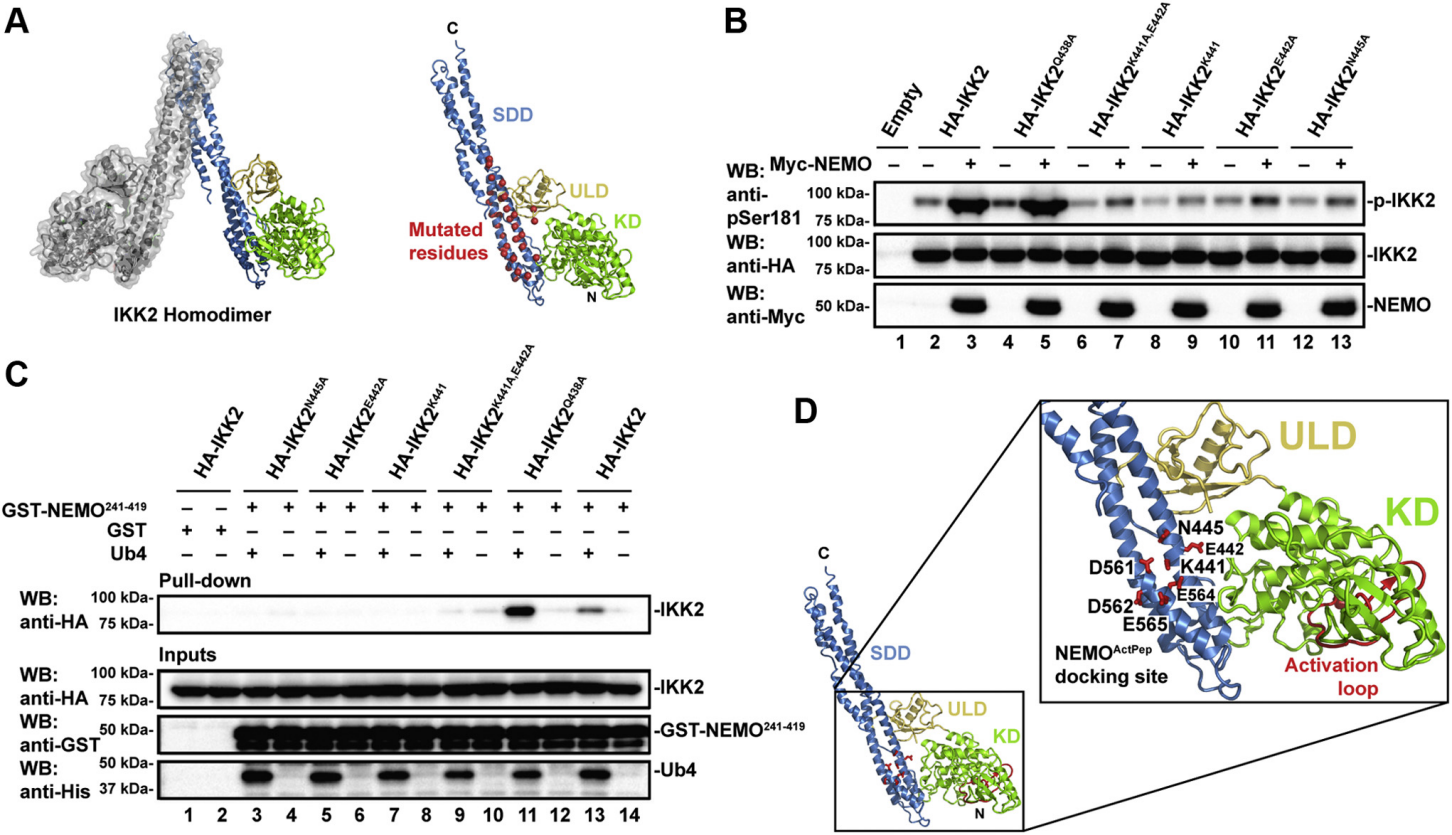
**Identification of a docking site for NEMO**^**ActPep**^**.***A*, a ribbon diagram representation of the human IKK2 homodimer with one subunit in *gray* with semitransparent surface rendered and the other colored by domain. On the *right* is an individual IKK2 monomer with domains labeled and Cα positions of each mutated residue indicated by a *red sphere*. *B*, Western blot analysis of lysates from 293 cells transfected with native and mutant full-length human HA-IKK2. Mutation of residues Glu441, Lys442, or Asn445 to alanine weakens the polyubiquitin-dependent association of NEMO with IKK2 (lanes 6–13). *C*, Western blot analysis of GST pull downs (*top panel*) from native and mutant HA-IKK2-transfected 293 cell lysates in the presence of linear tetraubiquitin and NEMO^241–419^. Inputs containing lysates augmented with purified proteins are shown below. *D*, a close-up view of the locations of Lys441, Glu442, and Asn445 as well as nearby acidic Asp561, Asp562, Glu564, and Glu565 residues that constitute proposed docking site on IKK2 for NEMO^ActPep^. IKK2 domains are labeled, and the kinase activation loop is depicted in *red*. GST, glutathione-*S*-transferase; HA, hemagglutinin; IKK, inhibitor of κB kinase; NEMO^ActPep^, NF-κB essential modulator activation peptide.

Thirty-eight different residues of IKK2 were altered to generate a total of 38 unique (28 single-point mutants, nine double, and one triple) mutant protein constructs (Figs. 4*A* and S4*A*). Upon cotransfection with NEMO into HEK293T cells followed by anti-HA Western blot analysis of cellular extracts, several of the mutants showed protein expression defects suggesting that those mutations destabilized protein folding and caused them to be degraded in the cell (Fig. S4*B*). The activation status of the IKK2 mutants that displayed near normal levels of expression was then assessed by anti-pSer181 Western blot. Four of the mutants, Lys441Ala/Glu442Ala, Lys441Ala, Glu442Ala, and Asn445Ala, displayed significantly reduced levels of activation loop phosphorylation compared with wildtype IKK2 indicating that the corresponding residues are critical for IKK2 activation (Figs. 4*B* and S4*B*). Expression levels of these mutants are similar to that of wildtype IKK2 and, as the mutations are located within the exposed surface of the proximal SDD, the observed defects in activation loop phosphorylation are not likely because of global structural perturbations.

To test if these mutants are defective in binding to NEMO through the second site, we performed GST pull-down assays in which *E. coli*-expressed GST-NEMO^241–419^ was mixed with whole-cell extracts of HEK293T cells expressing the IKK2 mutant proteins from transfected plasmids, both in the presence or in the absence of Ub4. Mutation of residues Lys441, Glu442, and Asn445 led to defects in binding (Figs. 4*C* and S4*C*). An IKK2 protein bearing mutation of a nearby residue, Gln438Ala, displayed no defects in phosphorylation of the activation loop and showed even stronger than native-like binding to GST-NEMO^241–419^ in the presence of Ub4, thus serving as a control. Further experiments are needed to understand the basis for the increased activation loop phosphorylation observed with this mutant protein.

The proximity of Lys441, Glu442, and Asn445 on the surface of the proximal SDD of IKK2 could indicate that these residues constitute a site for the poly-Ub–dependent second interaction with NEMO. We have dubbed this surface patch the NEMO^ActPep^ “docking site” of IKK2 (Fig. 4*D*). Since NEMO^ActPep^ contains the basic Arg385–Arg386 motif, we envisioned the likely possibility that an acidic pocket in IKK2 near docking site residues 441, 442, and 445 might also be engaged in NEMO^ActPep^ binding. Two such pockets exist, one within the ULD (Asp383 and Asp385) and the other in the SDD (Asp561, Asp562, Glu564, and Glu565). No defects on IKK2 activation loop phosphorylation in the presence of NEMO were observed upon mutation of either Asp383 or Asp385 to alanine (Fig. S4*D*). The effect of mutation of IKK2 SDD residues Glu561, Glu562, and Glu564 to alanine could not be assessed since these mutations resulted in loss of IKK2 expression.

### NEMO^ActPep^ disrupts IKK2 activation in cells

**Figure 5:**
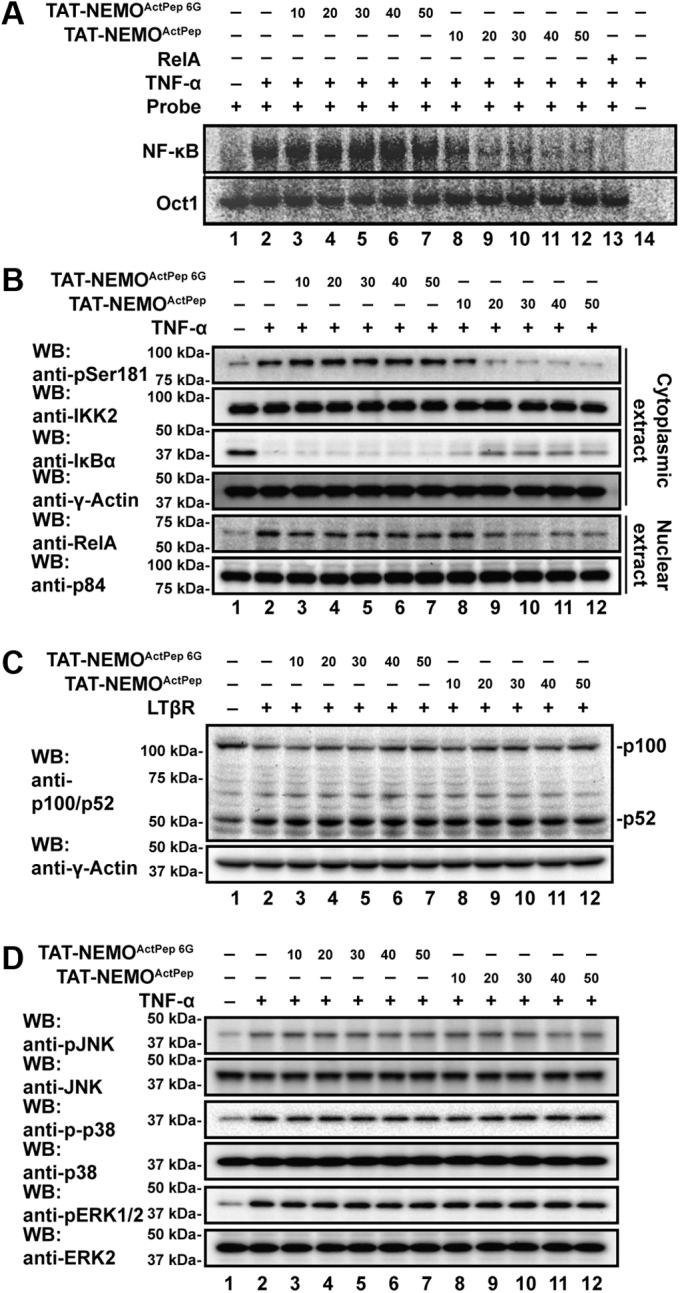
**NEMO**^**ActPep**^**specifically inhibits canonical NF-κB signaling.***A*, autoradiography of EMSA with nuclear extracts from HeLa cells treated with TNF-α after preincubation with increasing concentrations (10–50 μM) of either TAT-NEMO^ActPep^ or TAT-NEMO^ActPep 6G^. TAT-NEMO^ActPep^ disrupts NF-κB–dependent shift of radiolabeled probe (lanes 8–12, *upper panel*) in a dose-dependent manner, whereas control TAT-NEMO^ActPep 6G^ does not (lanes 3–7). *Lower panel* shows that binding of the constitutive nuclear transcription factor Oct1 to its probe is not affected by TAT-NEMO^ActPep^. *B*, Western blot analysis of cytoplasmic (*upper four panels*) and nuclear extracts (*lower two panels*) from TNF-α-treated HeLa cells after preincubation with increasing concentrations of either TAT-NEMO^ActPep^ or TAT-NEMO^ActPep 6G^. IKK2 activation loop phosphorylation and consequent IκBα degradation and NF-κB RelA subunit nuclear localization are significantly diminished in the presence of TAT-NEMO^ActPep^ (lanes 9–12). *C*, Western blot analysis indicates no change in the extent of p100 processing in response to activation of the noncanonical NF-κB pathway in HeLa cells with lymphotoxin β receptor (LTβR) after pretreatment with increasing concentrations of TAT-NEMO^ActPep^ (lanes 3–7) and TAT-NEMO^ActPep 6G^ (lanes 8–12). *D*, Western blot analyses reveal that preincubation with either TAT-NEMO^ActPep^ or TAT-NEMO^ActPep 6G^ has no effect upon levels of MAP kinase phosphorylation in TNF-α-treated HeLa cells. MAP, mitogen-activated protein; NEMO^ActPep^, NF-κB essential modulator activation peptide; TNF-α, tumor necrosis factor alpha.

We were intrigued by the ability of such a small segment (residues 384–389) of human NEMO to mediate interaction with and promote activation loop phosphorylation of IKK2. Thus, we explored the potential of a peptide derived from this region to function as a modulator of canonical NF-κB signaling through IKK2 in cells. HeLa S3 cells were pretreated with either TAT-NEMO^ActPep^ or control TAT-NEMO^ActPep 6G^ for 1 h prior to stimulation with 10 ng tumor necrosis factor alpha (TNF-α) for 15 min. NF-κB activity was measured by EMSA using nuclear extracts and radiolabeled κB DNA from the immunoglobulin kappa light chain gene (Ig-κB) as the probe (Fig. 5*A*). At 20 μM TAT-NEMO^ActPep^, the amount of shifted probe was significantly reduced, and at 40 μM peptide concentration, nuclear NF-κB DNA-binding activity was nearly abolished (Fig. S5*A*). This reduction was not observed in cells treated with the mutant peptide.

We further verified the inhibitory effects of TAT-NEMO^ActPep^ on canonical NF-κB signaling in HeLa cells by assessing IKK2 activation loop phosphorylation, IκBα degradation, and NF-κB RelA/p65 subunit subcellular localization. Consistent with our observations of decreased nuclear NF-κB DNA-binding activity by EMSA, treatment with TAT-NEMO^ActPep^ blocked IKK2 phosphorylation, IκBα proteolysis, and nuclear translocation of RelA (Fig. 5*B*). Treatment with the control peptide showed no such effects.

We next tested if the observed effect is universal across species and cell types. The effect of TAT-NEMO^ActPep^ was tested in RAW 264.7 murine macrophage cells treated with 100 ng/ml purified bacterial lipopolysaccharide (LPS) for 2 h. Again, NF-κB DNA binding and RelA subunit nuclear localization were both inhibited as were phosphorylation of the IKK2 activation loop and degradation of IκBα. The mutated peptide did not show such effects (Fig. S5*B*). EMSA analysis of LPS-treated RAW 264.7 cell nuclear lysates revealed inhibition of NF-κB activity after preincubation with TAT-NEMO^ActPep^ relative to TAT-NEMO^ActPep 6G^ to a similar degree as that observed in HeLa cells (Fig. S5*C*). We also analyzed the effect of TAT-NEMO^ActPep^ on NF-κB DNA binding in mouse embryonic fibroblast (MEF) cells by EMSA. Again, TAT-NEMO^ActPep^ but not TAT-NEMO^ActPep 6G^ inhibited NF-κB activation in response to TNF-α (Fig. S5*D*). It is important here to note that similar experiments in which a version of NEMO^ActPep^ lacking the cell penetrating N-terminal HIV-1 TAT sequence was applied together with K16ApoE synthetic transporter peptide yielded similar results, confirming that the TAT peptide is not responsible for the observed ability of NEMO^ActPep^ to competitively interfere with NEMO–IKK2 second-site binding and inhibit canonical NF-κB signaling (Fig. S6) (29, 30).

We also tested if TAT-NEMO^ActPep^ has any effect on noncanonical NF-κB signaling. This alternative NF-κB signaling pathway is activated in response to stimulation of specific cytokine receptors and leads to activation of IKK1 and consequent processing of the p100 precursor to the mature NF-κB subunit p52 (31, 32). We did not observe any effect of TAT-NEMO^ActPep^ or TAT-NEMO^ActPep 6G^ on processing of p100 in response to engagement of lymphotoxin-β receptor (LTβR), suggesting involvement of TAT-NEMO^ActPep^ specifically in canonical NF-κB signaling (Fig. 5*C*). To further investigate the possible involvement of TAT-NEMO^ActPep^ in other signaling pathways, we tested the effect of the peptide on mitogen-activated protein kinase (MAPK) activation by monitoring the phosphorylation status of c-Jun N-terminal kinase (JNK), extracellular signal–regulated kinase 2 (Erk2), and p38 upon TNF-α treatment of HeLa cells. As evidenced by Western blot with phospho-specific antibodies, each of the three MAPKs becomes phosphorylated in response to TNF-α treatment, as expected. Neither TAT-NEMO^ActPep^ nor TAT-NEMO^ActPep 6G^ influenced these phosphorylation processes (Fig. 5*D*). We conclude that TAT-NEMO^ActPep^ specifically inhibits IKK2 among signaling kinases in response to TNF-α treatment of HeLa cells and disrupts canonical signaling through IκBα to NF-κB.

### NEMO^ActPep^ does not display cellular toxicity common to IKK inhibitors

The major problem encountered with ATP-competitive small-molecule inhibitors of IKK2 is their toxicity (33). One such compound, MLN120B, has been extensively studied in mice and was shown to cause septic shock because of enhanced levels of prointerleukin (IL)-1β processing and to promote neutrophilia (34, 35). This compound has also been observed to block B-cell and T-cell proliferation (36). These observations have contributed to the belief that complete inhibition of IKK2 catalytic activity *via* selective high-affinity ATP-competitive drugs is not a beneficial therapeutic strategy.

**Figure 6:**
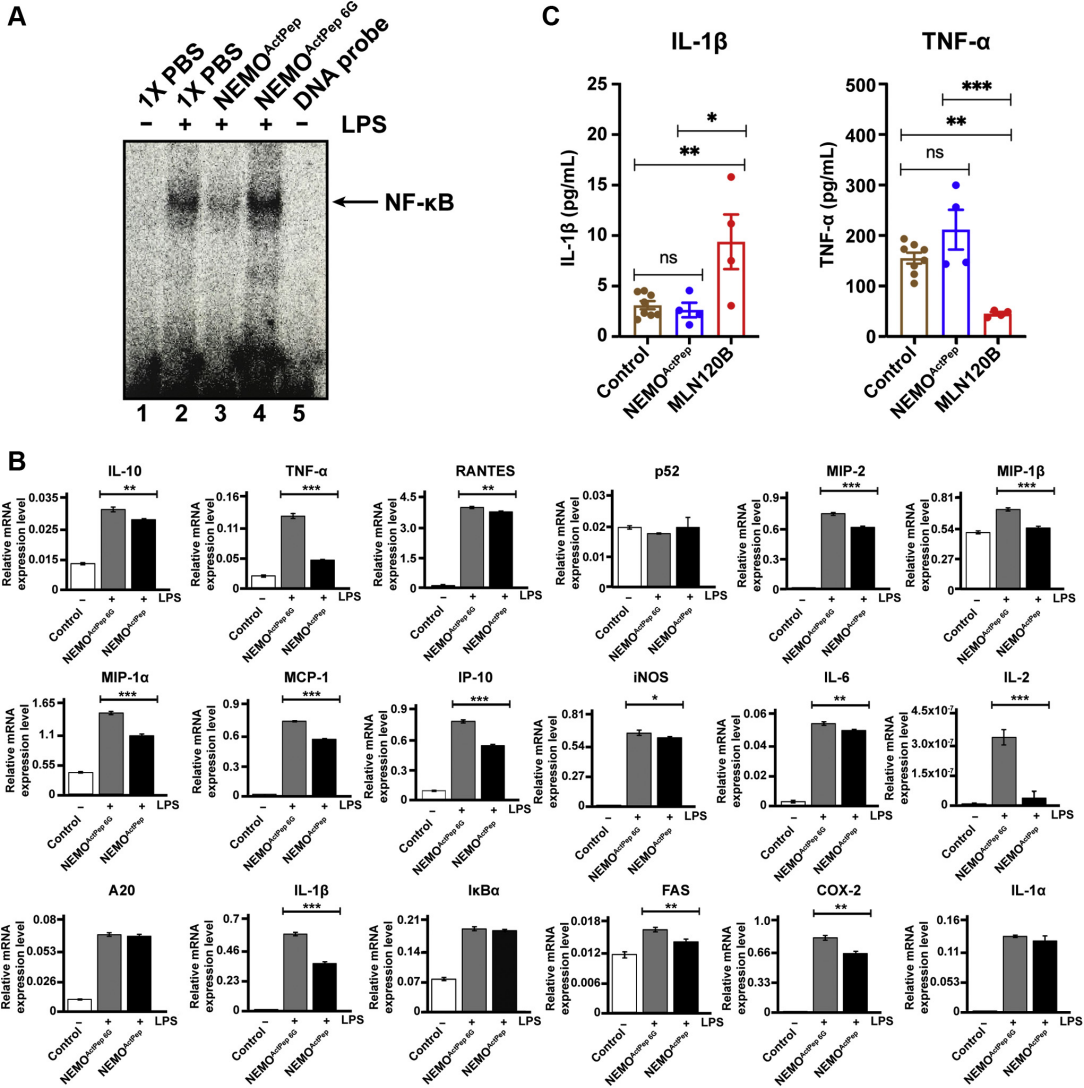
**NEMO**^**ActPep**^**does not display the toxicity profile associated with ATP-competitive IKK2 inhibitor compound MLN120B.***A*, autoradiography of EMSA with nuclear extracts from BMDM cells treated with LPS after preincubation with 50 μM of either TAT-NEMO^ActPep^ or TAT-NEMO^ActPep 6G^. TAT-NEMO^ActPep^ disrupts NF-κB–dependent shift of the radiolabeled probe (lane 3), whereas control TAT-NEMO^ActPep 6G^ does not (lane 4). *B*, the effect of TAT-NEMO^ActPep^ pretreatment on relative mRNA levels of select NF-κB target genes as measured by RT–qPCR of BMDM cells induced by LPS. Data were compared by unpaired *t* test. ∗*p* < 0.05; ∗∗*p* < 0.01; and ∗∗∗*p* < 0.001. *C*, protein levels of cytokines IL-1β and TNF-α in the supernatant of cultured BMDM cells preincubated with or without 100 μM TAT-NEMO^ActPep^ or MLN120B prior to TNF-α stimulation. BMDM, bone marrow–derived macrophage; IKK, inhibitor of κB kinase; IL-1β, interleukin 1β; LPS, lipopolysaccharide; NEMO^ActPep^, NF-κB essential modulator activation peptide; qPCR, quantitative PCR; TNF-α, tumor necrosis factor alpha.

We tested the toxicity of NEMO^ActPep^ in comparison to ATP-competitive inhibitors *ex vivo* using bone marrow–derived macrophage (BMDM) cells, a murine primary cell line. Consistent with our previous observations, EMSA revealed inhibition of NF-κB activation in response to LPS by pretreatment with TAT-NEMO^ActPep^ but not TAT-NEMO^ActPep 6G^ (Fig. 6*A*). We next analyzed mRNA transcript levels by RT–quantitative PCR in LPS-induced BMDM cells and observed that NEMO^ActPep^ treatment results in significant inhibition of some, but not all, NF-κB-regulated genes (Fig. 6*B*). This unique pattern of NF-κB-regulated gene expression could be due to low-level activity of residual nuclear NF-κB, which is sufficient to activate genes with strong κB sites and/or through the actions of other compensatory transcription factors such as AP1. These observations strongly suggest that NEMO^ActPep^ disrupts NF-κB signaling to a milder extent and against a select subset of NF-κB response genes.

Next, we tested the potentially toxic effects of a high concentration (100 μM) dose of NEMO^ActPep^ on IL-1β production in BMDMs without stimulating the cells. For comparison, we also treated BMDM cells with 100 μM MLN120B. ELISA analysis revealed that NEMO^ActPep^ failed to induce production of IL-1β. As reported, however, MLN120B treatment significantly induced production of mature IL-1β in these cells (Fig. 6*C*, *left panel*). The peptide also had no effect on TNF-α expression, whereas MLN120B reduced basal levels of TNF-α (Fig. 6*C*, *right panel*). These results suggest that disruption of canonical NF-κB signaling with NEMO^ActPep^ does not promote toxic levels of IL-1β as opposed to one well-studied ATP-competitive inhibitor.

## Discussion

It is well established that the IKK complex is the central hub of canonical NF-κB signaling initiated from a wide variety of diverse inducing signals. It has also been shown that IKK2 and NEMO are the essential components of the IKK complex that are required for canonical NF-κB signaling and that K63-linked and linear/M1-linked poly-Ub chains act through NEMO as intracellular inducers of IKK2 catalytic activity (37). The present study was motivated by our interest in understanding the mechanism through which NEMO and linear poly-Ub promote IKK2 activation, which is chemically defined by phosphorylation at two serines (Ser177 and Ser181) present within the activation loop of IKK2. In order to appreciate how changes to IKK complex structure and dynamics are associated with its activation, a brief discussion on the structural and dynamic features of both the IKK2 and NEMO subunits is essential.

**Figure 7:**
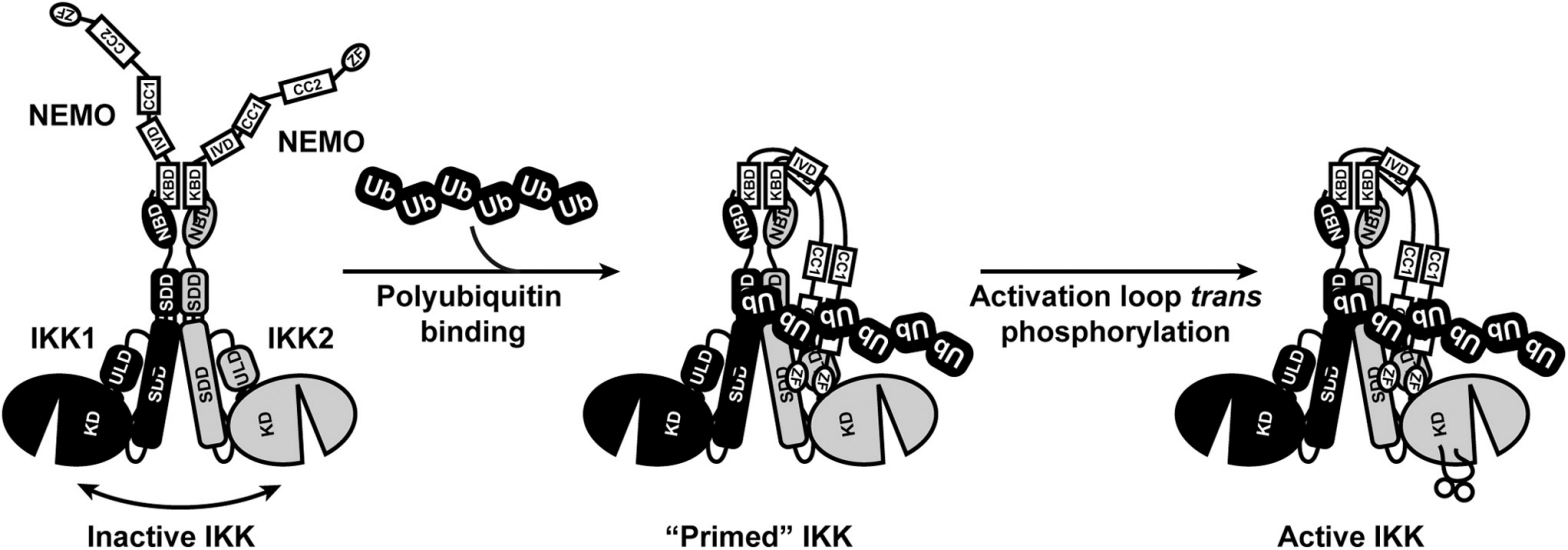
**Schematic representation of IKK2 subunit stabilization in response to linear polyubiquitin binding to NEMO subunits within the IKK complex.** The two NEMO subunits of the IKK complex heterotetramer associate *via* parallel homotypic coiled-coil interactions upon binding of linear polyubiquitin. This alters NEMO structure and dynamics and promotes second site interaction with IKK2 stabilizing the complex in an open conformation that is primed for activation *via* transphosphorylation of its activation segment. IKK, inhibitor of κB kinase; NEMO, NF-κB essential modulator.

IKK2 has four structurally characterized functional domains. Beginning from the N-terminal end of the protein, these are the KD, ULD, SDD, and NBD (Fig. 1*A*). The first three adopt folded structures and are connected to one another through noncovalent contacts forming the conserved “IKK-like kinase” core structure (Fig. 4*A*) (14, 15, 16, 17, 38, 39). The C-terminal NBD is highly dynamic, and its main function is to mediate stable interaction with the N-terminal KBD of NEMO, thereby holding the complex together. NEMO in turn is composed of an interrupted series of alpha-helical imperfect CC segments (Fig. 1*A*). Most of these CC segments require binding of accessory factors to stabilize their dimerization. For example, the N-terminal KBD of NEMO binds the IKK2 NBD to form IKK_2_–NEMO_2_ heterotetramers in solution. The central Ub-binding CC2 domain of NEMO, which spans residues 259 to 360, can form homodimers on its own, though a NEMO fragment containing both the CC2 and contiguous regions destabilizes CC2 homodimerization suggesting that neighboring portions of NEMO may antagonize dimerization of one another (40, 41). Located C-terminal to the CC2 domain of NEMO is the polypeptide sequence QRRSPP (amino acid residues 384–389) that we have identified as mediating the linear poly-Ub–dependent second interaction between NEMO and IKK2. Based on data presented in this study, we propose that, upon binding to M1-linked linear poly-Ub, the C-terminal portion of NEMO undergoes structural change exposing the NEMO^ActPep^ sequence for interaction with IKK2 (Fig. 7). Biophysical experimental evidence in support of gross conformational change of NEMO in response to linear poly-Ub binding in solution has been reported previously (42).

We report that the portion of NEMO that contains the NEMO^ActPep^ sequence binds to an exposed “docking site” on the surface of the IKK2 SDD near the KD–ULD. Although the IKK2 docking site for NEMO^ActPep^ is yet to be fully characterized, it is clear from our *in vitro* mutagenesis and binding data that the QRR tripeptide at NEMO amino acids 384 to 386 contributes significantly to the overall binding energy of the interaction. Therefore, we propose a mechanism for linear poly-Ub–dependent activation of IKK2 in which free heterotetrameric IKK complexes, each composed of two catalytic subunits as IKK1–IKK2 heterodimers in association with one NEMO_2_ homodimer, move through the cell cytoplasm as complex sensors that remain catalytically inactive on account of their inherent flexibility and dynamic character that restricts access of activating protein kinases to their activation loop serines. Association of the NEMO subunits with linear poly-Ub induces CC homodimer formation through the NEMO CC2 domains, which in turn prompts conformational change and association of the exposed NEMO activation peptide region with its docking site on the IKK2 subunit. This second interaction between NEMO and the catalytic IKK subunits stabilizes the IKK1–IKK2 heterodimer in an open conformation that is amenable to activation loop transphosphorylation and thus promotes IKK2 kinase catalytic activity (Fig. 7). Furthermore, interaction of the region of NEMO bearing the NEMO^ActPep^ sequence with the proximal SDD of IKK2 places the C-terminal zinc finger domain of NEMO, which has been shown previously to interact with the substrate IκBα protein, within close proximity of the portion of IKK2 that has also been shown to bind IκBα (14, 43). It remains to be determined whether this ligand-dependent kinase stabilization is a general mechanism through which NEMO serves as a versatile integrator of diverse cellular signals or if it is a specific consequence of its interaction with linear poly-Ub.

In the present study, we further explored the potential of an isolated NEMO^ActPep^ peptide to function as an effective inhibitor of IKK2 activation. We found that NEMO^ActPep^ is a highly specific inhibitor of the IKK2 activation pathway. Consistent with its dependence on a unique interaction between NEMO and IKK2, NEMO^ActPep^ does not block MAPK phosphorylation or IKK1 *via* the noncanonical NF-κB activation pathway. As a central process in inflammatory gene expression, IKK2-dependent induction of NF-κB transcriptional activity has been identified as a key contributor to numerous inflammatory disease syndromes including inflammation-induced cancers (44). Therefore, the canonical NF-κB signaling pathway has long been considered an attractive target for drug development. However, several promising small-molecule inhibitors could not be developed into drugs because of their toxicity. In those cases, complete abrogation of IKK2 activity *via* ATP-competitive binding and potential off target effects appeared to be the main cause for toxicity. We are encouraged by the novelty of NEMO^ActPep^ and the specificity it displays for canonical NF-κB signaling, though it remains to be seen whether NEMO^ActPep^ elicits significant off-target effects. Since the length of the peptide that confers inhibition is relatively short, its conversion to small molecule *via* peptidomimetic chemical approaches is a possible route for generation of novel lead compounds that function to inhibit IKK allosterically by interfering with the ability of linear Ub to trigger NEMO-dependent stabilization of catalytic subunit dimers. Identification of new non-ATP competitive small-molecule IKK2 inhibitor lead compounds have been reported recently, though it remains to be seen whether any of these functions through disruption of NEMO–IKK2 second-site binding (45).

## Experimental procedures

### Recombinant plasmid and baculovirus preparation

Human IKK2 complementary DNA (cDNA) (UniProt accession ID: O14920) was graciously provided by the laboratory of M. Karin (University of California San Diego School of Medicine). Full-length IKK2 was amplified by PCR and cloned in pFastBacHTb (Invitrogen) vector within BamHI and NotI sites in frame with an N-terminal hexahistidine-tobacco etch virus (TEV) cleavage site tag. Gene fragments corresponding to human IKK2^11–669^ and IKK1^10–667^ were amplified by PCR and subcloned into pFastBacHTb and pFastBacHTa, respectively. For site-directed mutagenesis, codons corresponding to S177 and S181 were mutated to E and K44 to M by PCR-based introduction of deoxyoligonucleotide primers harboring the mutations according to the Q5 site-directed mutagenesis protocol (New England Biolabs). Recombinant baculovirus production, amplification, and titer optimization were carried out in Sf9 insect cell suspensions as previously described (27).

Full-length human NEMO (UniProt accession ID: Q9Y6K9) and deletion mutants (NEMO^250–365^, NEMO^250–419^, and NEMO^111–419^) were subcloned individually into the NdeI and BamHI restriction sites of the pET15b vector in frame with an N-terminal hexahistidine tag. Methionine-linked Ub4 chain was subcloned into the BamHI and NotI restriction sites of the pET24d vector giving rise to an N-terminal hexahistidine tag followed by TEV protease recognition sequence.

The GST-NEMO fusions were constructed by subcloning the full-length cDNA and deletion variants NEMO^241–350^, NEMO^241–375^, NEMO^241–283^, NEMO^241–390^, NEMO^241–419^, and NEMO^111–419^ into the BamHI and NotI sites of pGEX4T-2 (GE Healthcare Life Sciences) in frame with an N-terminal GST tag followed by a TEV protease recognition sequence. Codons corresponding to Q384–R386 or Q384–P389 in the N-terminal GST-fused NEMO^241–419^ backbone were mutated to encode for G by PCR with base changes incorporated in the oligonucleotide primers.

For cellular assays, full-length human IKK2 cDNA was subcloned with an N-terminal HA tag into pRCCMV-HA (Clontech) vector. Several mutants (total of 38; 28 single, nine double, and one triple) of human IKK2 in which select residues were changed to A, S, or G were prepared by PCR with base changes incorporated in the primers. Full-length human NEMO was cloned as a Myc-tagged version into pCDNA-3.1 (Invitrogen). Residues 384 to 386 or 384 to 389 of the NEMO in pcDNA-3.1 were mutated G by PCR with base substitutions incorporated into the oligonucleotide primers.

### Cell culture and reagents

HeLa, HEK293T, and RAW 264.7 cells were obtained from American Type Culture Collection, and MEF cells were a gift from A. Hoffmann (University of California Los Angeles). Glutathione–agarose beads and Ni–nitrilotriacetic acid (NTA) agarose beads were purchased from BioBharati LifeScience Pvt Ltd. Mouse anti-HA antibody was purchased from BioLegend. Rabbit anti-NEMO, rabbit anti-IKK2, rabbit anti-His, rabbit anti-Ub, rabbit anti-Myc, rabbit anti-γ-actin, and rabbit anti-ERK2 were purchased from BioBharati LifeScience Pvt Ltd. Mouse anti-p84 was purchased from GeneTex. Mouse anti-GST, rabbit anti-p65/RelA, and rabbit anti-IκBα were from Santa Cruz Biotechnology. Rabbit anti-p100/p52 was a gift from N. Rice (National Cancer Institute; Frederick, MD). Rabbit anti–phospho-SAPK (stress-activated protein kinase)/JNK (Thr183/Tyr185), rabbit anti-SAPK/JNK, rabbit anti–phospho-p38 MAPK (Thr180/Tyr182), rabbit anti-p38 MAPK, rabbit anti–phospho-p44/42 MAPK (Erk1/2) (Thr202/Tyr204), and rabbit anti–phospho-IKKα/β (Ser177/181) were purchased from Cell Signaling. Horseradish peroxidase–conjugated anti-rabbit and antimouse secondary antibodies were from BioBharati LifeScience Pvt Ltd as was mouse TNF-α. LPS was purchased from Sigma. Mouse LTβR was purchased from Abcam.

### Peptides

The 17-mer NEMO^ActPep^ oligopeptide and the mutant NEMO^ActPep 6G^ peptide with 6 G residues, with and without diglycine-linked N-terminal TAT peptides, were synthesized by Bon Opus Biosciences. Peptides were characterized by matrix-assisted laser desorption ionization mass spectrometry and analytical reverse-phase high pressure liquid chromatography analysis. Peptides were dissolved in 1× PBS to stocks of between 2 and 10 mM.

### Protein expression and purification

All His-tagged NEMO proteins were expressed in Rosetta (DE3) *E. coli* cells (MilliporeSigma). Cultures of 1 l in LB media with 100 μg/ml ampicillin were grown at 37 °C to absorbance of 0.2 at 600 nm before induction with 0.2 mM IPTG (BioBharati LifeScience Pvt Ltd) and stirring at 150 rpm for 22 °C for 16 h. Cells were harvested by centrifugation at 3000*g* for 10 min (Beckman Coulter), and cell pellets were lysed by sonication (VWR Scientific) on ice in 200 ml of lysis buffer (20 mM Tris–HCl [pH 8.0], 500 mM NaCl, 10% w/v glycerol, 10 mM imidazole, 0.2% Triton X-100, 1 mM PMSF, and 5 mM β-mercaptoethanol). Lysates were clarified by centrifugation at 15,000 rpm for 45 min. Supernatants containing soluble proteins were then applied to a 1 ml Ni–NTA agarose column that was pre-equilibrated with lysis buffer. Bound proteins were washed with 200 ml wash buffer (lysis buffer with 40 mM imidazole) and eluted in 10 ml elution buffer (lysis buffer containing 150 mM NaCl and 250 mM imidazole).

Sf9 insect cells from 1 l suspension cultures were harvested by centrifugation at 3000*g* for 10 min at 4 °C and lysed by sonication in 100 ml of lysis buffer (25 mM Tris–HCl, pH 8.0, 200 mM NaCl, 10 mM imidazole, 10% w/v glycerol, and 5 mM β-mercaptoethanol). The lysate was clarified by centrifugation twice at 18,000 rpm for 45 min at 4 °C. Pre-equilibrated Ni–NTA agarose resin was added at a ratio of 1 ml of resin slurry/liter of lysed cell culture, and the mixture was incubated on a rotator at 4 °C for 3 h. The Ni beads were pelleted at 1000 rpm for 2 min in a swinging bucket centrifuge rotor. Supernatant was carefully decanted, and the protein-bound resin was resuspended with wash buffer (lysis buffer containing 30 mM imidazole) and incubated at 4 °C on a rotator for 2 min. The Ni beads were pelleted again and decanted (wash 1). This was repeated until the last wash fraction contained 0.01 to 0.1 mg/ml of protein (Bio-Rad Protein Assay). Elution buffer (lysis buffer containing 250 mM imidazole) was added, and eluted fractions were collected and stored at −80 °C.

Recombinant N-terminal GST-tagged NEMO proteins were expressed in Rosetta (DE3) by growing cells to an absorbance of 0.2 at 600 nm followed by induction with 0.2 mM IPTG for 16 h at 22 °C. Cells were lysed in 200 ml lysis buffer (25 mM Tris [pH 8.0], 500 mM NaCl, 0.1% [v/v] Triton X-100, 10% [v/v] glycerol, 1 mM EDTA, 1.0 mM PMSF, and 5 mM β-mercaptoethanol) and sonicated. The lysate was clarified by centrifugation at 15,000 rpm for 45 min at 4 °C. The supernatant was loaded onto a Glutathione–agarose resin column pre-equilibrated with lysis buffer at 4 °C. After wash with 200 ml lysis buffer, protein was eluted with elution buffer (25 mM Tris [pH 8.0], 150 mM NaCl, 0.1% [v/v] Triton X-100, 10% [v/v] glycerol, 5 mM β-mercaptoethanol, and 10 mM glutathione). Eluted fractions were collected and stored at −80 °C.

### Fractionation by SEC

Purified individual full-length His-tagged IKK2, NEMO, and Ub4 proteins as well as NEMO–Ub4 complexes were subjected to gel filtration with a Superose6 Increase 10/300 GL size-exclusion column (GE Healthcare) on an NGC Liquid Chromatography System (Bio-Rad). The column was equilibrated, and samples were run in buffer (25 mM Tris–HCl, pH 8.0, 250 mM NaCl, 2 mM DTT, and 5% glycerol) at a flow rate of 0.2 ml/min at 22 °C. Peak fractions were collected and analyzed by Coomassie-stained SDS-PAGE.

### Whole-cell extract preparation and nuclear–cytoplasmic fractionation

HEK293T cells were cultured in Dulbecco's modified Eagle's medium (DMEM) supplemented with 10% (v/v) fetal bovine serum and 1% (v/v) penicillin/streptomycin/glutamine. At 70 to 80% confluence, cells were transiently transfected with empty, HA-IKK2WT, or HA-IKK2 mutant plasmids using polyethyleneimine (PolySciences). Cells were harvested 48 h post-transfection. To prepare whole-cell extracts, cells were lysed in radioimmunoprecipitation assay (RIPA) buffer (10 mM Hepes–KOH [pH 7.8], 250 mM NaCl, 1 mM EDTA [pH 8.0], 0.5% [v/v] NP-40, 0.2% [v/v] Triton X-100, 2 mM DTT, 20 mM β-glycerophosphate, 10 mM NaF, 0.1 mM Na_3_VO_4_, 1 mM PMSF, and 1× protease inhibitor cocktail) with gentle shaking for 1 h at 4 °C. Lysed cells were centrifuged at 13,000 rpm for 20 min at 4 °C, and supernatants containing the whole-cell protein extracts were quantified by Bio-Rad protein assay.

To prepare nuclear and cytoplasmic protein extracts, HeLa, MEF, and RAW 264.7 cells were lysed in buffer containing 4.3 mM Na_2_HPO_4_, 1.47 mM KH_2_PO_4_ (pH 7.4), 137 mM NaCl, 2.7 mM KCl, 1 mM DTT, 0.05% (v/v) NP-40, and 1× protease inhibitor cocktail for 10 min on ice and spun at 3000 rpm at 4 °C for 10 min. The supernatant containing the cytoplasmic fraction was quantified by Bio-Rad protein assay to determine the total amount of protein. Pellets were resuspended in nuclear extraction buffer (25 mM Tris–HCl [pH 7.5], 420 mM NaCl, 10% glycerol, 0.2 mM EDTA, 1 mM DTT, 0.5 mM PMSF, and 1× protease inhibitor cocktail) once and then subjected to three lysis cycles (freeze at −80 °C and thaw at 37 °C). Finally, samples were centrifuged at 13,000 rpm at 4 °C for 20 min, and supernatants containing the soluble nuclear fraction were measured by the Bio-Rad protein assay to determine the total amount of protein. Nuclear and cytoplasmic extracts were aliquoted and kept at −80 °C.

### Cell culture, stimulation, and peptide treatment of cells

HeLa, MEF, and RAW 264.7 cells were cultured in DMEM containing 10% fetal bovine and 2 mM l-glutamine serum supplemented with penicillin and streptomycin, at 37 °C in a humidity incubator with 5% CO_2_. RAW 264.7 cells were seeded and allowed to adhere for 24 h and then treated with NEMO^ActPep^ or NEMO^ActPep 6G^ at different concentrations 60 min before the LPS challenge (100 ng/ml). After 2 h, nuclear and cytoplasmic extracts were collected. HeLa and MEF cells seeded for 24 h were then treated with NEMO^ActPep^ or NEMO^ActPep 6G^ at different concentrations 60 min before the TNF-α challenge (10 ng/ml) for 15 min and then nuclear and cytoplasmic extracts were collected. MEF cells were treated with NEMO^ActPep^ or NEMO^ActPep 6G^ at different concentrations 1 h before the LTβR challenge (300 ng/ml). After 24 h, whole-cell extracts were collected and aliquoted and kept at −80 °C for further experiments.

### Western blot analysis

Equivalent amounts of protein from cell extracts were separated by SDS-PAGE and transferred to polyvinylidene fluoride membranes (Millipore). The membrane was blocked with 5% bovine serum albumin for 1 h at room temperature, and then the membrane was incubated with the primary antibodies overnight at 4 °C. Antibodies for IKK2, His, HA, Myc, GST, Ub, IκBα, phospho-IKK2, p65/RelA, p84, γ-actin, p100/52, phospho-ERK, phospho-JNK, phospho-p38, ERK, JNK, and p38 were used for detecting multiple specific protein targets. After binding of an appropriate secondary antibody coupled to horseradish peroxidase, the immunoreactive bands were visualized by enhanced chemiluminescence substrate.

### *In vitro* GST pull-down assay with purified recombinant proteins

Glutathione–agarose beads equilibrated with the binding buffer (25 mM Tris–HCl [pH 7.5], 150 mM NaCl, 1 mM EDTA, 0.5% NP-40, 0.55% Triton X-100, 5% glycerol, and 1 mM DTT). Purified recombinant N-terminal GST-NEMO fusion proteins were mixed with recombinant purified IKK2 proteins in the absence or the presence of Ub4. The mixtures were incubated in GST-binding buffer on a rotating platform for 1 h at 4 °C and then mixed with glutathione–agarose beads. After 2 h incubation at 4 °C, GST–fusion protein complexes bound to glutathione–agarose beads were washed four times with the binding buffer. Proteins bound to the beads were resuspended in SDS-PAGE loading buffer, resolved by SDS-PAGE, and analyzed by Western blot.

### *In vitro* pull-down assay from whole-cell extract

Proteins of HEK293T cells transfected with full-length human IKK2 or mutants were extracted using RIPA buffer. After centrifugation at 16,000*g* for 20 min, the supernatants were adjusted to the binding buffer (containing 1.5 mU hexokinase) and incubated on a rotating platform for 1 h at 4 °C. These whole-cell extracts were then incubated with *E. coli*-expressed GST-fusion NEMO in the absence or in the presence of Ub4 on a rotating platform for 2 h at 4 °C. The beads were centrifuged at 1000*g* for 2 min and washed four times in binding buffer. Proteins bound to beads were resuspended in SDS loading buffer, resolved by SDS-PAGE, and analyzed by Western blot.

For peptide *in vitro* pull-down assay, proteins of HEK293T cells expressing full-length human IKK2 were extracted using the same lysis buffer described previously. Whole-cell lysates were mixed with *E. coli*-expressed GST-NEMO with or without NEMO^ActPep^ or mNEMO^ActPep^ at different concentrations in the absence or in the presence of Ub4 and incubated on a rotating platform for 2 h at 4 °C. The beads were then washed four times in the binding buffer. Proteins bound to beads were resuspended in SDS-PAGE loading buffer, separated by SDS-PAGE, and analyzed by Western blot.

### Cell-based *in vitro* kinase activity assay

HEK293T cells were cultured in DMEM supplemented with 10% (v/v) fetal bovine serum and 1% (v/v) penicillin/streptomycin/glutamine. Cells were transiently transfected with empty vector, wildtype, or mutant HA-IKK2, or cotransfected with Myc-NEMO, 3G, or 6G mutant plasmids using polyethyleneimine following the manufacturer’s protocol. After being transfected for 48 h, cells were harvested and lysed in RIPA buffer for 1 h at 4 °C. Then, cells were centrifuged at 13,000 rpm for 15 min at 4 °C, and supernatants containing the whole-cell protein extracts were quantified by Bio-Rad protein assay to determine. Proteins were added SDS-PAGE loading buffer and heated to 95 °C for 5 min. Samples were resolved on 10% SDS-PAGE and analyzed by Western blot using antibody against phospho-Ser181 IKK2 with anti-HA and anti-Myc antibodies were used for loading controls. Protein expression was normalized against native sequence IKK2.

### EMSA

EMSA was performed using recombinant full-length NF-κB RelA homodimer as a positive control as previously described (46). Briefly, Ig-κB probe was radiolabeled with ^32^P-γ-ATP (6000 Ci/mmol; 10 μCi/μl) and incubated with nuclear lysates for 20 min at room temperature in binding buffer (10 mM Tris–HCl [pH 7.5], 10% [v/v] glycerol, 1% [v/v] NP-40, 1 mM EDTA, and 0.1 mg/ml salmon sperm DNA). Samples were separated in 4% nondenaturing polyacrylamide gels in 24.8 mM Tris base, 190 mM glycine, and 1 mM EDTA buffer at 200 V for 1 h, and the gel was dried. The amount of protein in lysates was quantified by Bio-Rad protein assay. EMSA analysis with quantitative densitometry and signal intensity were quantitated using ImageJ software (47). All EMSA experiments were performed in triplicate.

### BMDM cell culture

With the approval of the University of California San Diego and Veteran Affairs San Diego Institutional Animal Care and Use Committee, BMDM cells were isolated from 6- to 8-week-old C57BL/6J mice (Jackson Laboratory) followed by erythrocyte lysis with ammonium chloride and then seeded in 12-well plates at a concentration of 1 × 10^6^ cells/ml. Cultured cells were differentiated to macrophages with culture media containing monocyte-colony stimulating factor (10 ng/ml). After 7 days of differentiation, BMDM cells were treated with TNF-α (10 ng/ml) or LPS (100 g/ml) with or without TAT-NEMO^ActPep^, TAT-NEMO^ActPep 6G^, or MLN120B.

### Extraction of total RNA and cDNA synthesis

BMDM cells were pretreated with or without 50 μM TAT-NEMO^ActPep^ or TAT-NEMO^ActPep 6G^ and then stimulated with or without LPS (100 ng/ml) overnight. Cells in the negative control sample were treated with 1× PBS. The total RNA was extracted using TRIzol Reagent (Thermo Fisher Scientific) according to the manufacturer’s instructions. The yield and purity of RNA were determined by absorbance spectrophotometry, and RNA samples with absorbance of 1.8 to 2.0 at 260/280 ratio were analyzed. First-strand cDNA was synthesized from 2 μg total RNA using 20 μl of Super Reverse Transcriptase MuLV Kit (BioBharati LifeScience Pvt Ltd) with random hexamer oligonucleotide primers and incubation at 42 °C for 3 min to protect total RNA from genomic DNA interference. Reverse transcription was performed using Super RT enzyme at 50 °C for 15 min. Experiments were performed in triplicate.

### Quantitative RT–PCR

For each reaction, 2 μl cDNA product was combined with SYBR Green (New England Biolabs) in a C1000 Touch thermocycler (Bio-Rad) using murine primers to IL-10, TNF-α, RANTES (Regulated upon Activation, Normal T Cell Expressed and Presumably Secreted), p52, macrophage inflammatory protein (MIP)-1α, MIP1β, MIP-2, monocyte chemoattractant protein 1, IP-10, inducible nitric oxide synthase, IL-6, IL-2, A20, IL-1α, IL-1β, IκBα, FAS, cyclooxygenase 2, and GAPDH. The reaction was performed in triplicate. The amplification conditions were as follows: 44 cycles at 95 °C for 15 s, 55 °C for 30 s, and 0.5 °C gradient increase from 60 to 95 °C. Quantitative mRNA expression data were normalized to the expression levels of GAPDH (dCt = Ct gene of interest − Ct GAPDH) and reported as relative mRNA expression (ddCt = 2^−(dCt sample − dCt control)^) or fold change.

### Quantitation of cytokines

BMDM cells were preincubated with or without 100 μM TAT-NEMO^ActPep^ or MLN120B for 24 h. Negative control cells were treated with 1× PBS or dimethyl sulfoxide at the same concentrations as those present in either TAT-NEMO^ActPep^ or MLN120B, respectively. IL-1β and TNF-α levels were measured from 20 μl supernatant of cultured BMDM cells using U-PLEX mouse cytokine assay kit (Meso Scale Diagnostics) following the manufacturer’s protocol. The absorbance of samples was measured spectrophotometrically. Experiments were performed in triplicate.

### Statistical analysis

Statistics were calculated with Prism 8 (version 8.4.3) software (GraphPad Software, Inc). Data were analyzed using unpaired two-tailed Student's *t* tests for comparison of two groups or one-way or two-way ANOVA for more than two groups followed by Tukey post hoc analysis if appropriate. All data are presented as mean ± SEM. *p* Value <0.05 was considered statistically significant in all analyses.

## Data availability

All data are contained within the article and supporting information.

## Supporting information

This article contains supporting information.

## Conflict of interest

G. G., T. H., T. B., and S. K. M. are the founders of Siraj Therapeutics. All other authors declare that they have no conflicts of interest with the contents of this article.
