# Supplementary material for: Regulatory subunit NEMO promotes polyubiquitin-dependent induction of NF-κB through a targetable second interaction with upstream activator IKK2
Source: J Biol Chem. 2022 Mar 24;298(5):101864. doi: 10.1016/j.jbc.2022.101864 (PMC9035715; doi:10.1016/j.jbc.2022.101864)
Supplement: Supplemental Figures S1–S6 [file mmc1.docx]

**SUPPLEMENTAL INFORMATION**


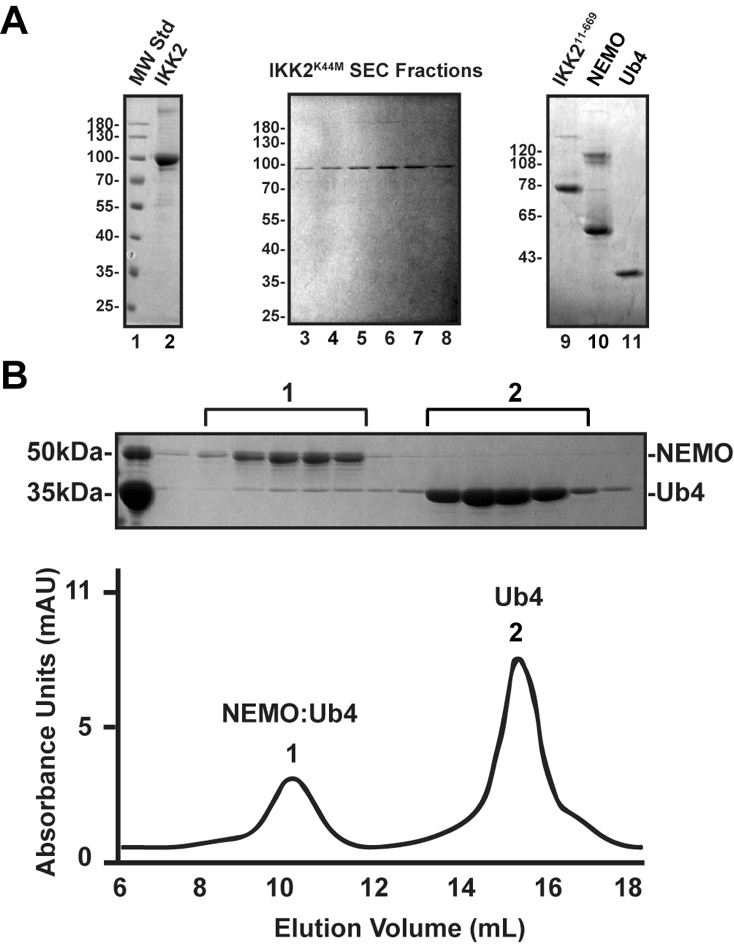


**Supplementary Figure 1.** A) Coomassie-stained SDS PAGE analysis of purified recombinant proteins employed in *in vitro* IKK2 *trans* auto-phosphorylation kinase assay: full length IKK2 (lane 2), IKK2^K44M^ (lanes 3-8), IKK2^11-669EE^ (lane 9), full length NEMO (lane 10) and linear tetraubiquitin (lane 11). B) Coomassie-stained SDS PAGE (above) and chromatogram of size exclusion chromatography (below) of a mixture of purified full length human NEMO and linear tetraubiquitin (Ub4). The complex elutes as peak 1 and free Ub4 is peak 2.


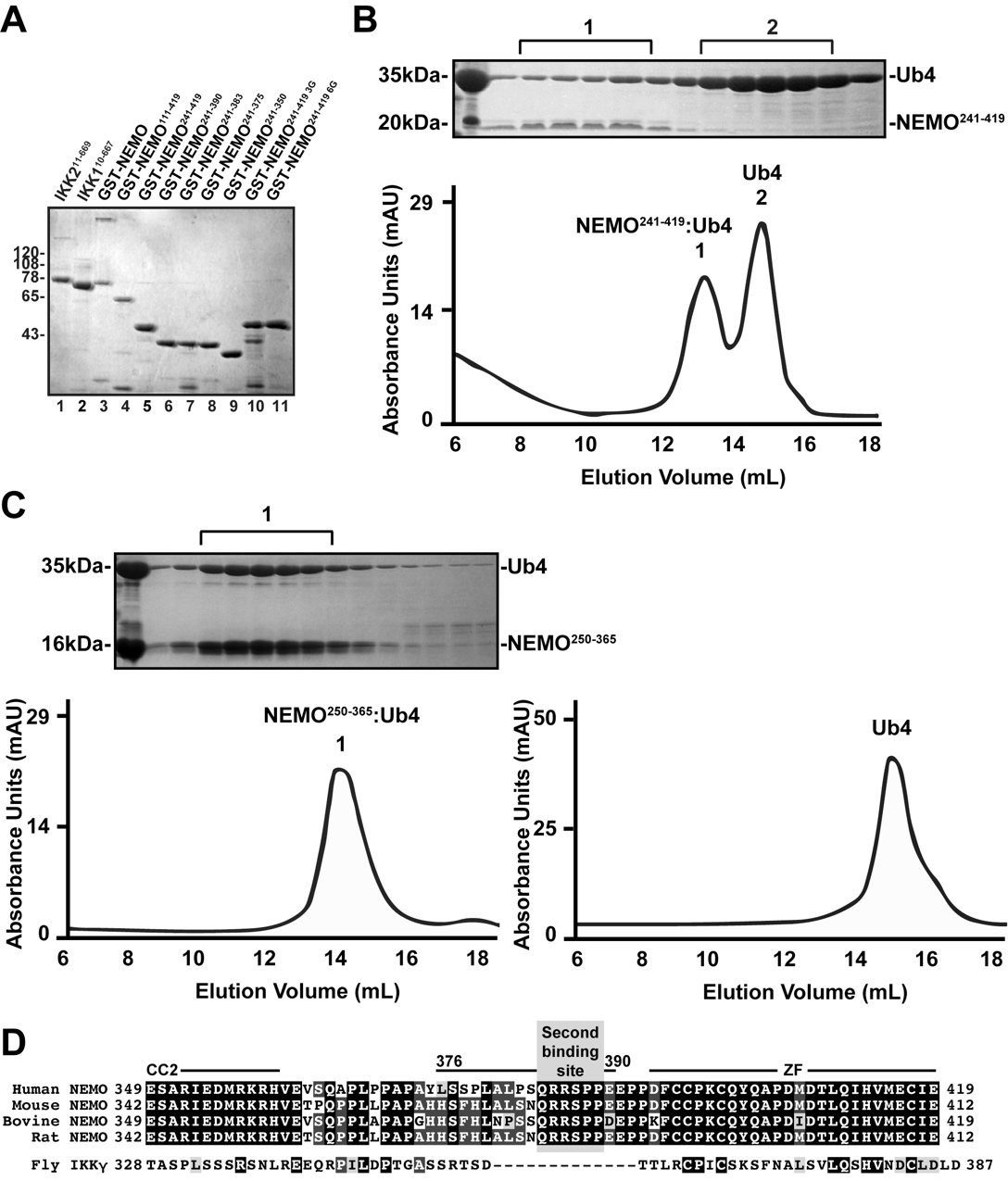


**Supplementary Figure 2.** A) Coomassie-stained SDS PAGE of purified recombinant IKK2 and GST-NEMO protein deletion constructs employed in mapping the secondary binding site on to residues 384-389 of human NEMO. B) Coomassie-stained SDS PAGE (above) and chromatogram of size exclusion chromatography (below) of a mixture of purified NEMO^241-419^ and linear tetraubiquitin (Ub4). The complex elutes as peak 1 and free Ub4 is peak 2. C) Coomassie-stained SDS PAGE (above) and chromatograms of size exclusion chromatography of a mixture of purified NEMO^250-365^ and linear tetraubiquitin (Ub4) (below left) and free Ub4 (below right). The complex elutes as peak 1 and free Ub4 is peak 2. D) Comparison of primary amino acid sequences from the C-terminal ends of mammalian NEMO proteins. The proposed second binding site (labeled) is a conserved sequence of six amino acids corresponding to human NEMO residues 384-389 within a proline-rich region linking the CC2 and ZF (both labeled). Identical residues are present in mouse, bovine, and rat NEMO proteins. The *Drosophila* IKKγ homolog, which has not been shown conclusively to be required for *Drosophila* IKKβ activation in response to linear polyubiquitin, lacks this motif.


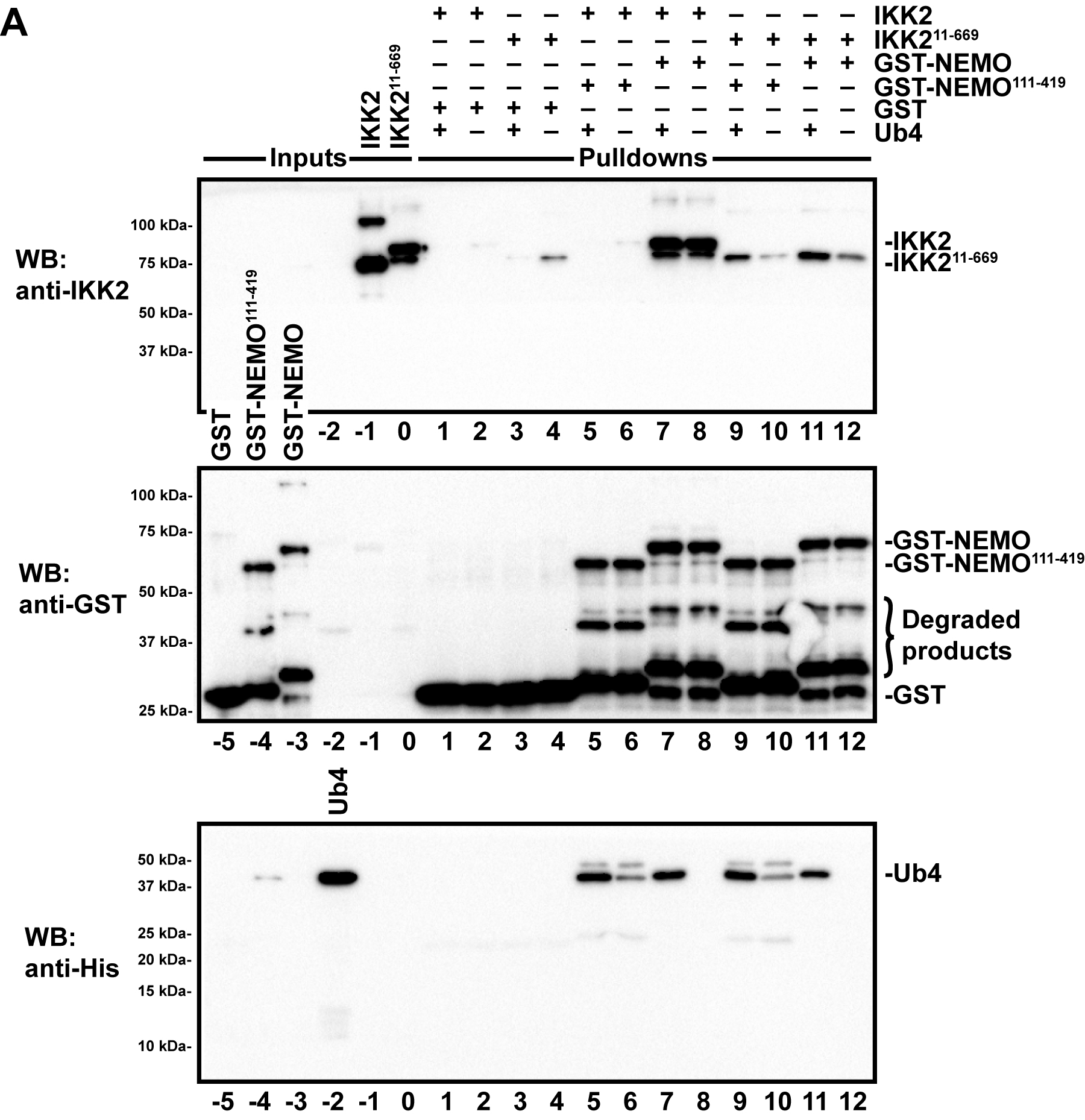


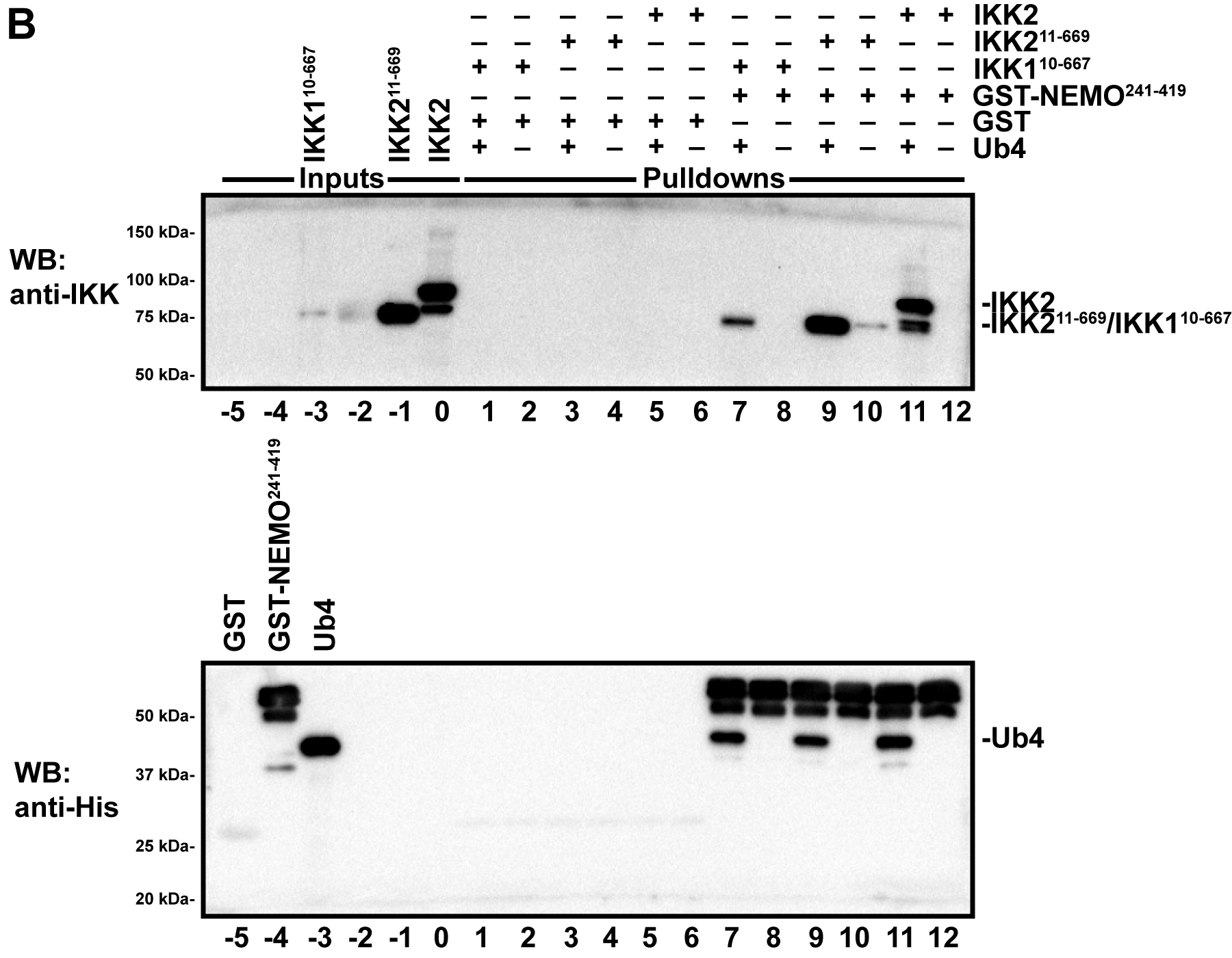


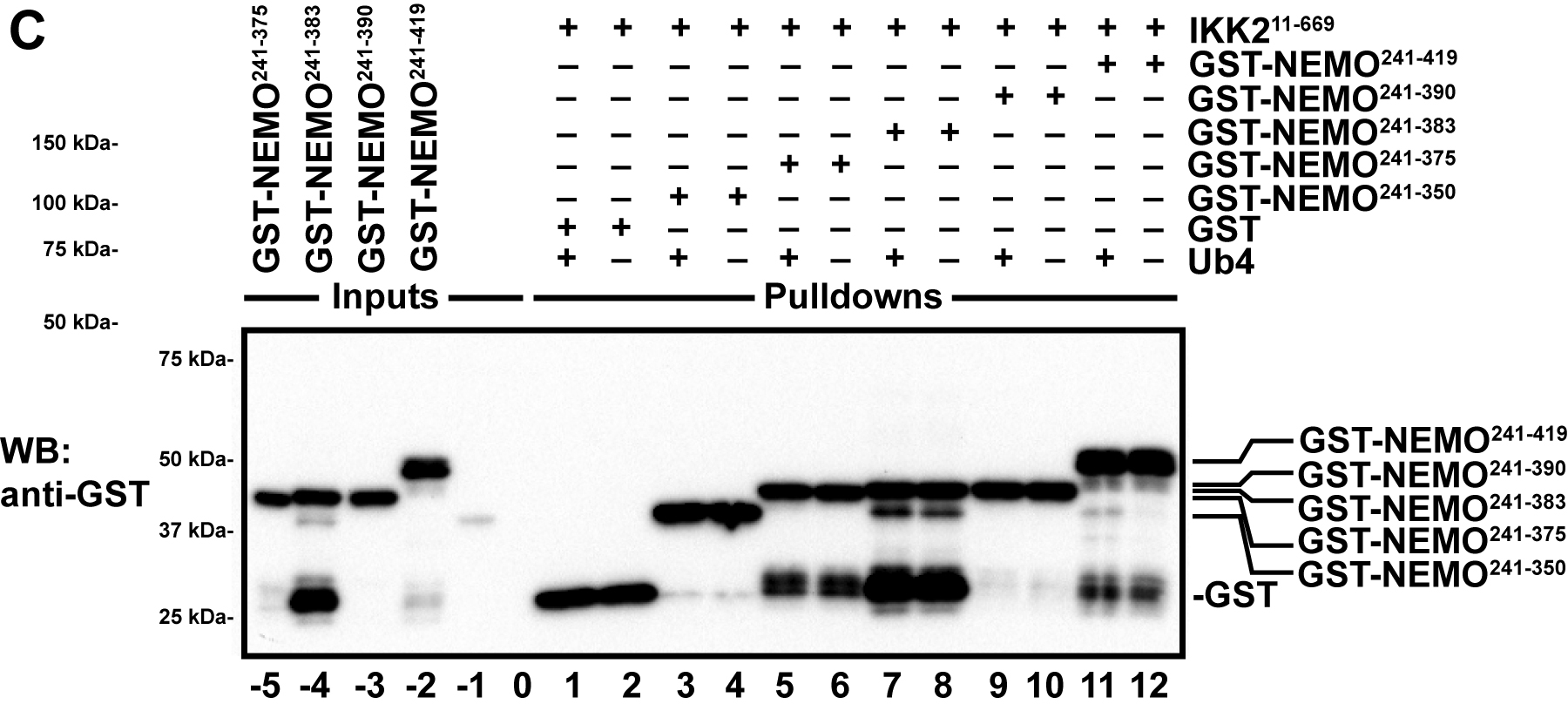


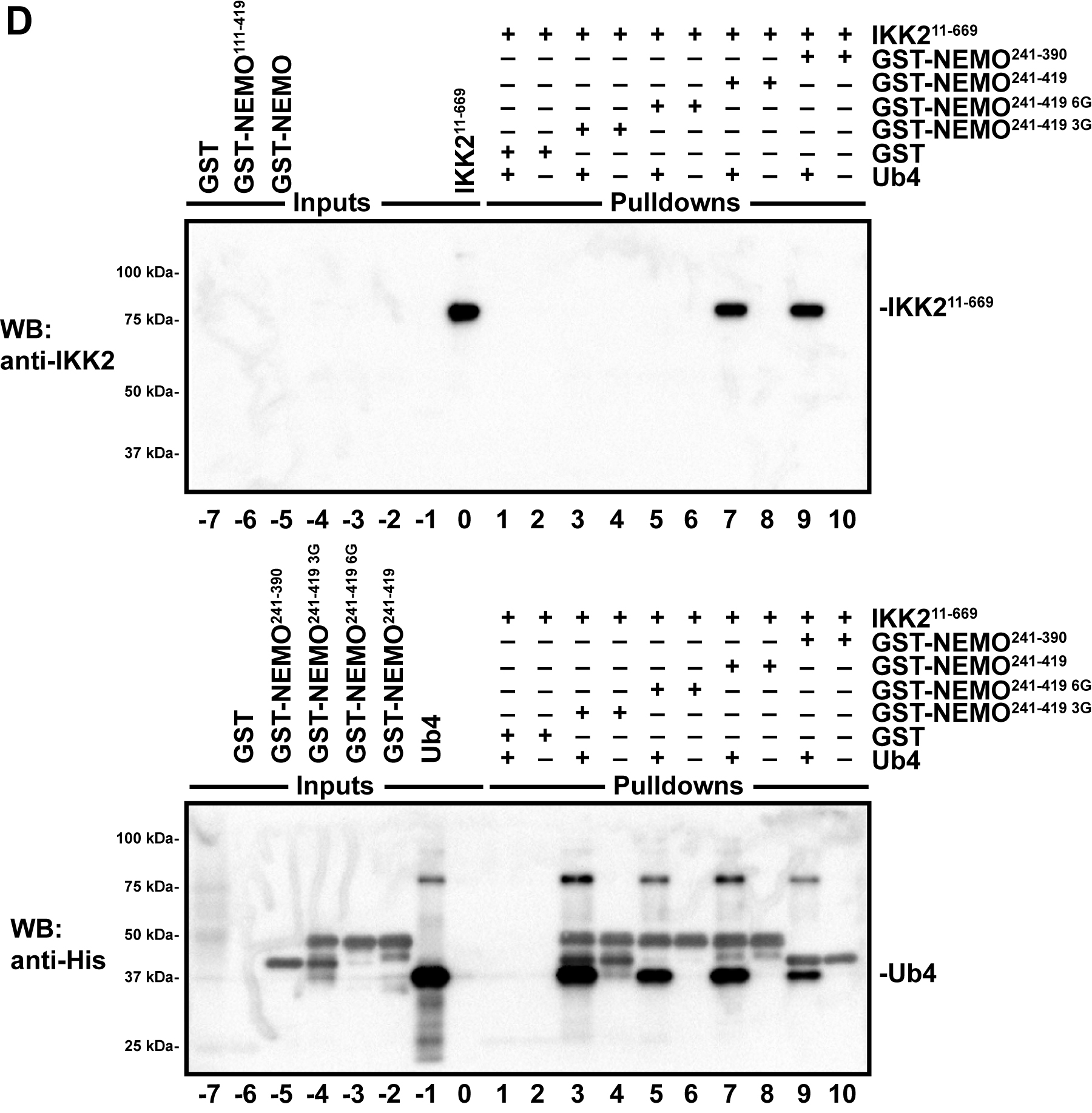


**Supplementary Figure 3.** Western blots for pull-down experiments in Figure 2. A-D correspond to the respective panels in Figure 2. Purified proteins used in the experiment are detected prior to GST-pull-down (an “Inputs”) in lanes -7 through 0. Lanes 1-12 are the same as in Figure 2 but the entire blots are shown. Excess GST proteins both in the input and pull-down lanes appear as non-specific “ghost” bands in anti-His blots in panels B and D.


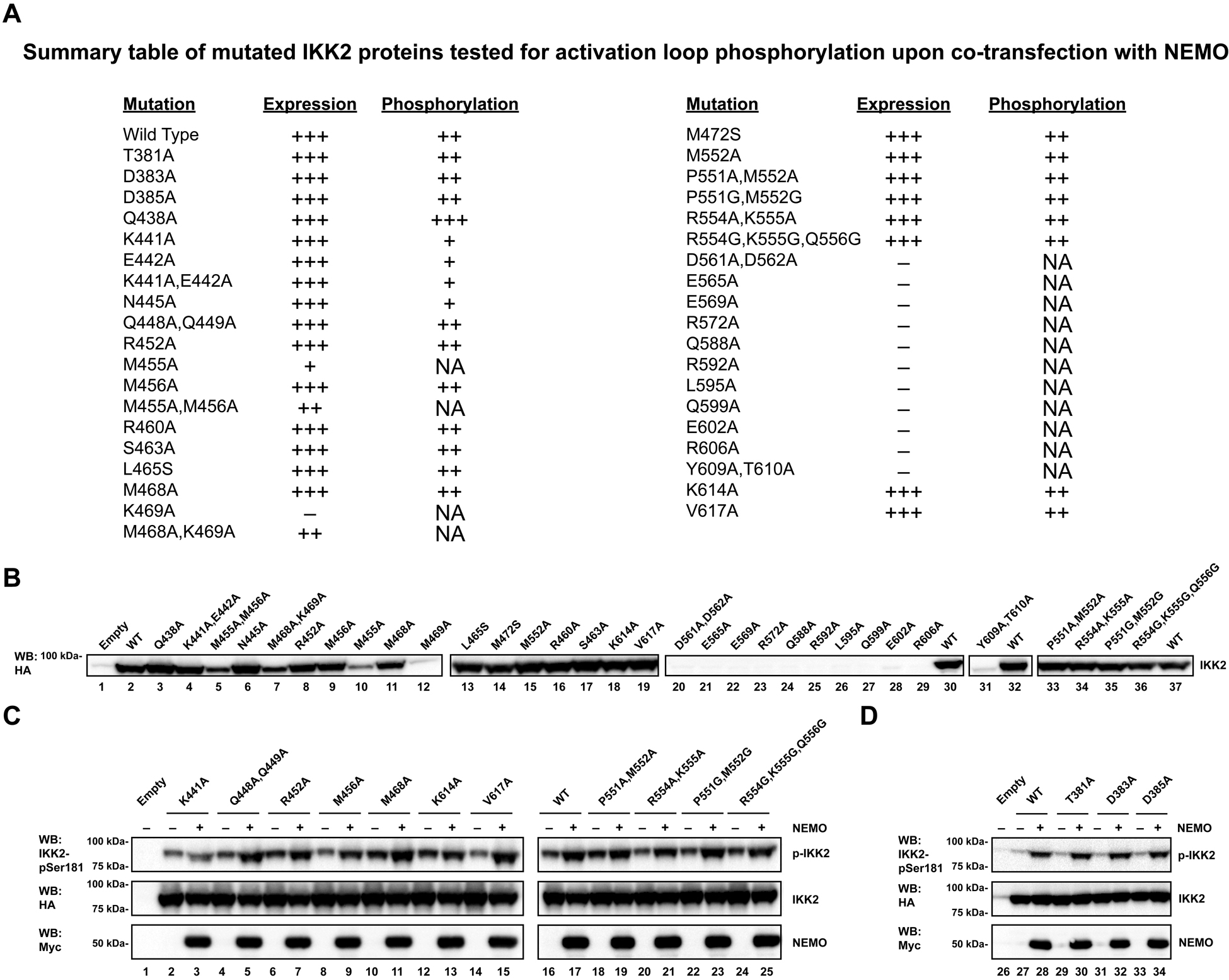


**Supplementary Figure 4.** A) A summary table of all mutant IKK2 proteins employed in mapping the second site of linear polyubiqutin-dependent interaction with NEMO including their observed expression levels and degree of activation loop phosphorylation upon co-transfection with NEMO. B) Western blot analysis monitoring expression of native (WT) and mutant human IKK2 proteins in transfected HEK293T cells. C) Western blot analysis monitoring the extent of IKK2 activation loop phosphorylation in HEK293T cells transfected with WT or mutant IKK2 in the absence (-) or presence (+) of co-transfected NEMO. D) Western blot analysis indicates that mutation of aspartic acid residues 383 and 385 to alanine does not interfere with the ability of IKK2 to become fully active when co-transfected with NEMO into HEK293T cells (lanes 31-34).


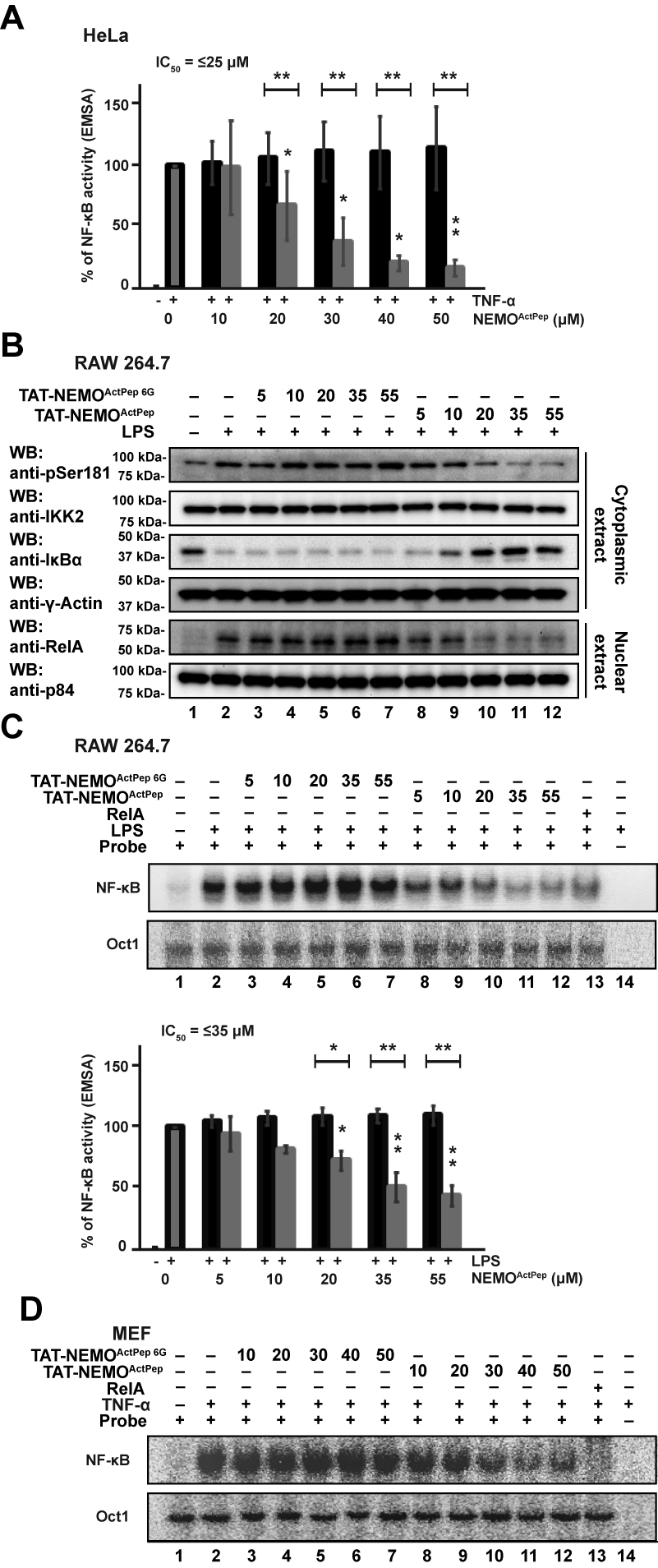


**Supplementary Figure 5.** A) Densitometry analysis of Ig-κB DNA binding by HeLa cell nuclear lysates as detected by EMSA (Figure 5A). Data were compared by unpaired *t*-test. *p < 0.05; **p < 0.01. B) Western blot analysis of cytoplasmic (upper four panels) and nuclear extracts (lower two panels) from LPS-treated RAW 264.7 cells after pre-incubation with increasing concentrations of either TAT-NEMO^ActPep^ or TAT-NEMO^ActPep 6G^. C) Autoradiography (above) of EMSA with nuclear extracts from RAW 264.7 cells treated with LPS after pre-incubation with increasing concentrations (5-55 μM) of either TAT-NEMO^ActPep^ or TAT-NEMO^ActPep 6G^. Densitometry analysis of Ig-κB DNA binding by RAW 264.7 cell nuclear lysates as detected by EMSA (below). Data were compared by unpaired *t*-test. *p < 0.05; **p < 0.01. D) Autoradiography of EMSA with nuclear extracts from MEF cells treated with TNF-α after pre-incubation with increasing concentrations (10-50 μM) of either TAT-NEMO^ActPep^ or TAT-NEMO^ActPep 6G^.


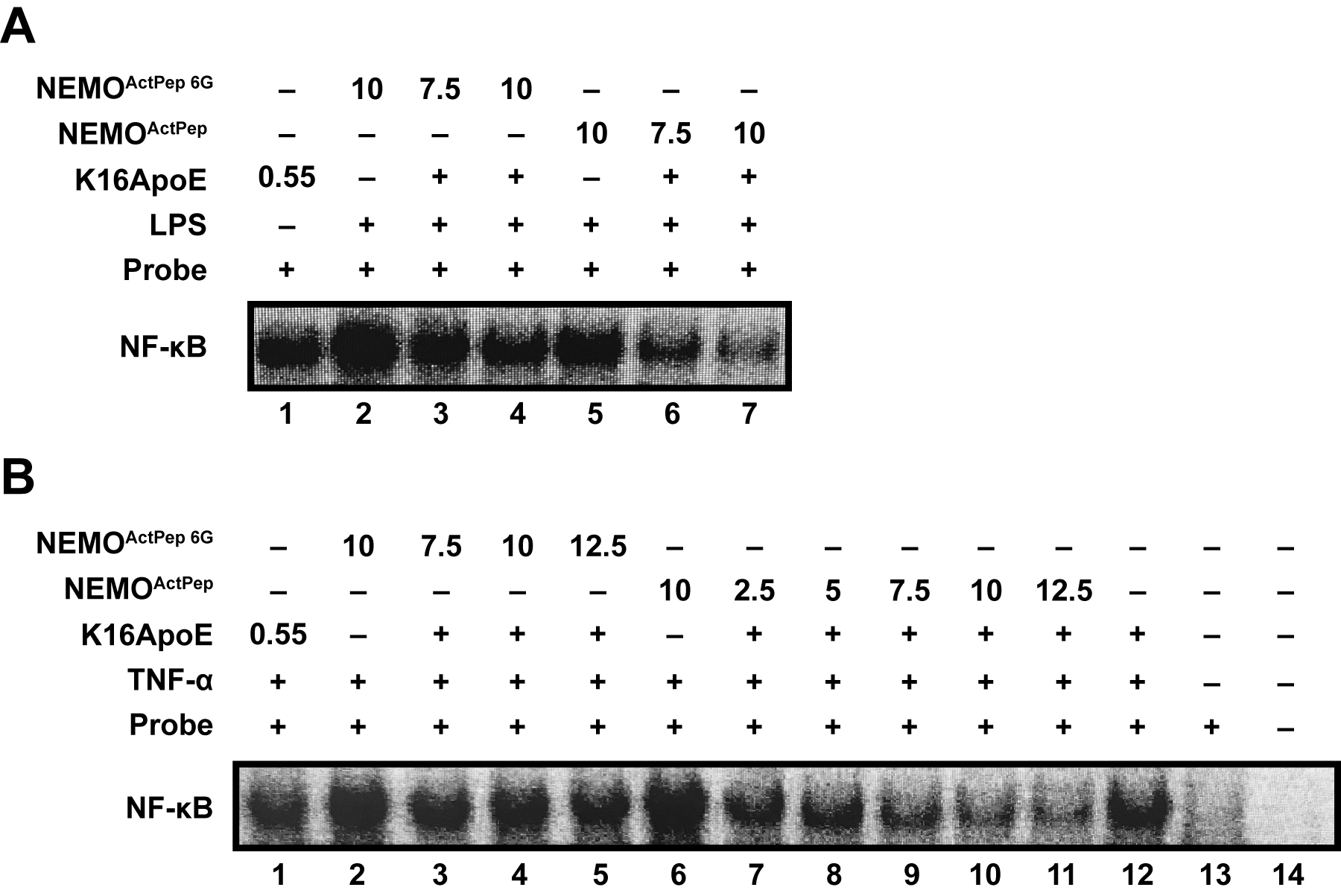


**Supplementary Figure 6.** Gel shift assays of κB DNA binding activity in purified nuclei from HeLa cells induced with either LPS (Panel A) or TNF-α (Panel B). In both experiments, cells were treated with versions of NEMO^ActPep^ and NEMO^ActPep 6G^ lacking the N-terminal HIV-1 TAT cell permeabilization peptide sequence. Rather, the peptides were introduced to cells in combination with the indicated amounts (in μM) of K16ApoE synthetic transport peptide. This shows that the inhibitor effect on NF-κB activity of NEMO^ActPep^ is not reliant upon the HIV-1 TAT peptide. EMSA of Ig-κB DNA binding by HeLa cell nuclear lysates and autodradiography was performed as described in Figure 5.
